# LRP6 Is a Functional Receptor for Attenuated Canine Distemper Virus

**DOI:** 10.1128/mbio.03114-22

**Published:** 2023-01-16

**Authors:** Vaiva Gradauskaite, Marine Inglebert, John Doench, Melanie Scherer, Martina Dettwiler, Marianne Wyss, Neeta Shrestha, Sven Rottenberg, Philippe Plattet

**Affiliations:** a Division of Neurological Sciences, Vetsuisse Faculty, University of Bern, Bern, Switzerland; b Institute of Animal Pathology, Vetsuisse Faculty, University of Bern, Bern, Switzerland; c Bern Center for Precision Medicine, University of Bern, Bern, Switzerland; d Graduate School for Cellular and Biomedical Sciences, University of Bern, Bern, Switzerland; e Genetic Perturbation Platform, Broad Institute of MIT and Harvard, Cambridge, Massachusetts, USA; Columbia University Medical Colleges

**Keywords:** attenuated CDV, CRISPR/Cas9 KO screen, LRP6, cell entry, receptor

## Abstract

Wild-type canine distemper virus (CDV) is an important pathogen of dogs as well as wildlife that can infect immune and epithelial cells through two known receptors: the signaling lymphocytic activation molecule (SLAM) and nectin-4, respectively. Conversely, the ferret and egg-adapted CDV-Onderstepoort strain (CDV-OP) is employed as an effective vaccine for dogs. CDV-OP also exhibits promising oncolytic properties, such as its abilities to infect and kill multiple cancer cells *in vitro*. Interestingly, several cancer cells do not express SLAM or nectin-4, suggesting the presence of a yet unknown entry factor for CDV-OP. By conducting a genome-wide CRISPR/Cas9 knockout (KO) screen in CDV-OP-susceptible canine mammary carcinoma P114 cells, which neither express SLAM nor nectin-4, we identified low-density lipoprotein receptor-related protein 6 (LRP6) as a host factor that promotes CDV-OP infectivity. Whereas the genetic ablation of LRP6 rendered cells resistant to infection, ectopic expression in resistant LRP6^KO^ cells restored susceptibility. Furthermore, multiple functional studies revealed that (i) the overexpression of LRP6 leads to increased cell-cell fusion, (ii) a soluble construct of the viral receptor-binding protein (solHOP) interacts with a soluble form of LRP6 (solLRP6), (iii) an H-OP point mutant that prevents interaction with solLRP6 abrogates cell entry in multiple cell lines once transferred into recombinant viral particles, and (iv) vesicular stomatitis virus (VSV) pseudotyped with CDV-OP envelope glycoproteins loses its infectivity in LRP6^KO^ cells. Collectively, our study identified LRP6 as the long sought-after cell entry receptor of CDV-OP in multiple cell lines, which set the molecular bases to refine our understanding of viral-cell adaptation and to further investigate its oncolytic properties.

## INTRODUCTION

As cancer remains one of the major causes of death in developed countries, immunotherapy using OVs has become an important field of research. OVs are able to specifically infect and lyse tumor cells, causing minimal damage to healthy tissue. Importantly, by inducing immunogenic cell death, OVs have been shown to generate a systemic anti-tumor immune response against tumor antigens (1). Various attenuated viruses are currently being tested as potential therapeutic agents *in vitro*, using animal models as well as human clinical trials (2). Three of those OVs (Oncorine, Talimogene Laherparepvec (T-VEC), and G47Δ (DELYTACT)) have already been approved for the use in human cancer therapy (3–5). The measles virus (MeV) and CDV are closely related viruses that belong to the same genus, *Morbillivirus*. The vaccine strain of MeV (Edmonston) not only has been employed in humans as one of the most efficient live-attenuated vaccines developed thus far but has also been extensively studied as a potential vector by which to treat human cancers (6–9). While wild-type (wt) MeV and CDV enter cells through SLAM and nectin-4 (N4) receptors (10–12), the attenuated MeV-Edmonston (Edm) strain has adapted, through multiple passages in cell cultures, to use membrane cofactor protein CD46 as an additional cell entry receptor (13). However, systemic MeV-Edm delivery may be limited by the fact that most cancer patients have already been vaccinated with this strain earlier in their lives. On the other hand, CDV naturally escapes neutralization by the sera of individuals immunized against MeV (14). Thus, MeV-Edm particles that are engineered to harbor the CDV glycoproteins have been designed to potentially bypass preexisting MeV immunity, which may be particularly attractive for systemic delivery protocols (14–17).

The vaccine strain CDV-OP originated from “Green’s distemperoid virus” that was serially passaged in ferrets (18) and then in chicken embryos (19), which resulted in the generation of an efficient live-attenuated vaccine for dogs (20). Interestingly, CDV-OP can infect multiple cells in cultures expressing neither SLAM nor N4 (21, 22). However, in contrast to MeV-Edm, the identity of the missing receptor (previously referred to as receptor X or “xR” [23]) remains to be determined. MeV and CDV encode two surface glycoproteins that are essential for cell entry: the receptor-binding (H) protein and the fusion (F) protein. Cell entry is initiated upon the engagement of the H protein to a cognate receptor that is expressed on target cells, which in turn activates the F protein. The fusion protein then undergoes a series of irreversible conformational changes, ultimately resulting in the formation of a fusion pore and the injection of the ribonucleocapsid into the cytoplasm of the host cell.

Genome-wide CRISPR knockout screening approaches have been successfully used to investigate virus-host interactions (24–27). Here, we conducted a genome-scale CRISPR/Cas9 KO screen in a canine mammary carcinoma cell line (P114) to identify the host factors that are required for CDV-OP infection. A sequencing analysis of enriched single-guide RNA (sgRNA) from pooled CDV-OP-resistant P114^KO^ cells identified the membrane-anchored LRP6 as a reproducible top hit. Using several orthogonal approaches, we validated LRP6 as the receptor X, which acts as a key host factor for CDV entry in a wide variety of cell types.

## RESULTS

### A genome-wide CRISPR/Cas9 KO screen identified LRP6 as a host factor for CDV-OP infection.

**FIG 1:**
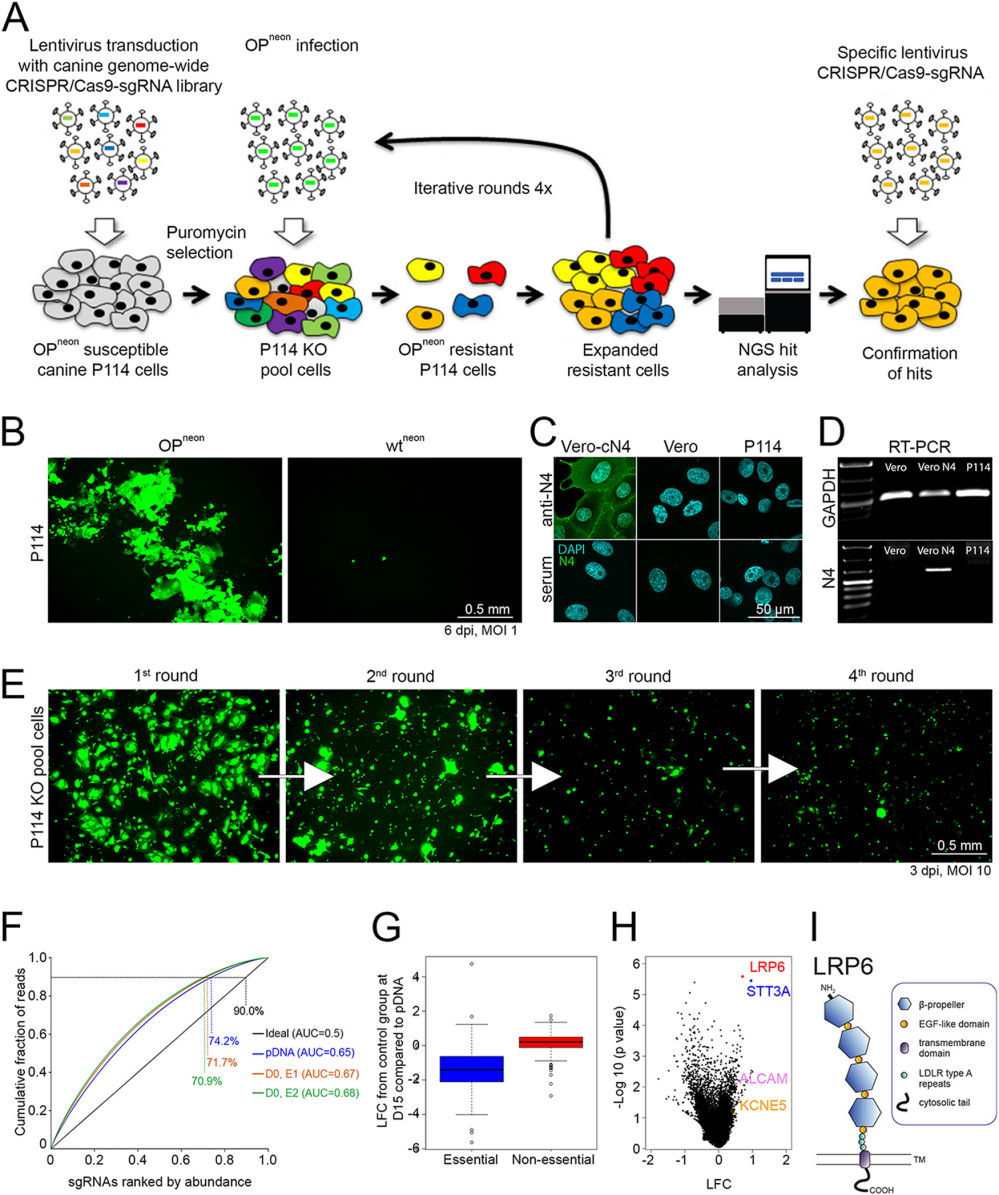
Genome-wide CRISPR/Cas9 Knock Out screen identifies potential host factors that are essential for CDV-OP infectivity. (A) Schematic outline of the canine genome-wide CRISPR/Cas9 KO screen. OP^neon^, mNeonGreen expressing CDV-OP strain. (B) Infection of canine mammary carcinoma P114 cells with CDV-OP (left panel) or CDV wild-type (right panel) expressing mNeonGreen (OP^neon^ and wt^neon^, respectively). (C) Assessment of cN4-expression in P114 cells by immunofluorescence (IF) analyses, using an anti-N4 antibody. (D) Investigation of cN4 (with GAPDH as a control) mRNA expression via reverse transcription-PCR. (E) Enrichment of cells acquiring CDV-OP-resistance through successive rounds of infection of P114 cells transduced with the canine genome-wide CRISPR library. (F) Cumulative distribution and area under the curve (AUC) analysis of the library representation. The representation was evaluated for the pDNA of the Beauty library (blue graph) as well as for the genomic DNA that was extracted from the D0 population (experiment 1 [E1], red/orange and experiment 2 [E2], green). An ideally distributed library is shown in black. The percentages indicate each library’s representation at 90% cumulative reads, and the AUC values are indicated in the legend. (G) Boxplot representing the log fold change (LFC) (D15, compared to the plasmid DNA of the library [pDNA], obtained for the control population) of genes known to be essential or nonessential. (H) Volcano plot representing the depleted (log fold change [LFC] < 0) and enriched (LFC > 0) genes after four rounds of infection with OP^neon^, compared to the control population. The LFC and *P* values were calculated from two independent replicates via a MAGeCK analysis. Each dot represents one gene for which at least two gRNAs (out of four) were used for the analysis. Selected hits are color-coded. (I) Schematic illustration of the LRP6 receptor, consisting of four β-propeller domains linked by epidermal growth factor (EGF)-like motives, low-density lipoprotein receptor (LDLR) class A repeats, a transmembrane domain, and a cytoplasmic tail.

We performed two independent, genome-wide CRISPR/Cas9 KO screens, following the same protocol, to the identify host factors that promote CDV-OP infection. To this aim, we initially designed a canine genome-scale library called “Beauty”, which consists of more than 80,000 unique single-guide RNA (sgRNA) targeting 20,039 protein-encoding genes (four sgRNA per gene). All of the genes are listed in Table S1. The library was subsequently cloned into a lentiviral-based expression construct (lentiCRISPRv2). To facilitate the screen, we employed an mNeonGreen (neon)-expressing CDV-OP recombinant virus (OP^neon^) that was engineered to deliver the reporter protein from an additional expression cassette that was cloned at the 3′ end of the genome (28) (Fig. 1A). We finally selected the canine mammary carcinoma cell line P114 (29) because (i) OP^neon^ mediates a lytic infection (Fig. 1B, left panel), (ii) a wild-type CDV strain (A75/17; wt^neon^) that exclusively employs SLAM and N4 as receptors did not infect those cells (Fig. 1B, right panel), (iii) canine N4-expression is below the detection limit as monitored by immunofluorescence (IF) as well as reverse transcription polymerase chain reaction (RT-PCR) assays (Fig. 1C and D), and (iv) SLAM is known to be expressed exclusively by immune cells and not epithelial cells, such as P114 (30).

10.1128/mbio.03114-22.4 TABLE S1 Genes targeted by the genome-wide canine CRISPR/Cas9 library and gRNAs counts (plasmid DNA pDNA, day 0 D0, CDV-treated condition, control [ctrl] for experiment 1 [E1] and experiment 2 [E2]). Download Table S1, TXT file, 2.3 MB. Copyright © 2023 Gradauskaite et al. 2023 Gradauskaite et al. https://creativecommons.org/licenses/by/4.0/ This content is distributed under the terms of the Creative Commons Attribution 4.0 International license.

Following the transduction of P114 cells with the genome-wide sgRNA library at a multiplicity of infection (MOI) of 1, the successfully transduced cells were selected with puromycin. We aimed to express the library at a coverage of 300 cells per sgRNA. A pool of these transduced cells was harvested at the beginning of the screen and is referred to as day 0 (D0). We then exposed the pool of puromycin-resistant cells to 4 successive rounds of OP^neon^ infections (MOI of 10), using an interval of 3 days, on average. Uninfected control cells were maintained in parallel. As expected, the number of neon-expressing cells decreased after each round of infection, indicating the successful enrichment of CDV-OP-resistant P114^KO^ cells (Fig. 1E). On day 15 (D15), cells were harvested, and extracted genomic DNA was sent for next-generation sequencing and subsequent analyses.

After data normalization, the distribution of the sgRNA counts remained similar between the original plasmid DNA (pDNA) and D0 for both experiments (Fig. 1F). We then calculated the log fold change (LFC) of each perturbation (sgRNA) for each sample (infected and control) and compared them to D0 via the MAGeCK (Model-based Analysis of Genome-wide CRISPR-Cas9 Knockout) software package (31). Moreover, when comparing the genes from the control population with essential genes that were previously detected in human cells (32), we found that the latter were also depleted in our experiment (LFC <0), which suggests a good library activity (Fig. 1G). Strikingly, the analysis of the sgRNAs of the OP^neon^-resistant cells at day 15, compared to the control population, showed a strong enrichment for two of the sgRNAs targeting the membrane-anchored LRP6 in both experiments (Fig. 1H). LRP6 (Fig. 1I) was determined as the strongest hit, using two independent algorithms: MAGeCK (31) and the gscreend R package (33). In addition to LRP6, Table S2 illustrates a list of additional hits that were identified via MAGeCK analysis as possible essential host factors that promote CDV-OP infection in P114 cells. Hence, our screen for CDV-OP resistance using the Beauty library yielded interesting hits that may contribute to our knowledge of how CDV-OP infects and kills its host cell.

10.1128/mbio.03114-22.5 TABLE S2 List of the first 100 ranked gene candidates that were identified in 2 experiments of the CRISPR/Cas9 screen. The analysis was run using MAGeCK software. CDV-treated group versus the control at D15. Download Table S2, DOCX file, 0.01 MB. Copyright © 2023 Gradauskaite et al. 2023 Gradauskaite et al. https://creativecommons.org/licenses/by/4.0/ This content is distributed under the terms of the Creative Commons Attribution 4.0 International license.

### LRP6 is an essential host factor in controlling CDV-OP infections.

**FIG 2:**
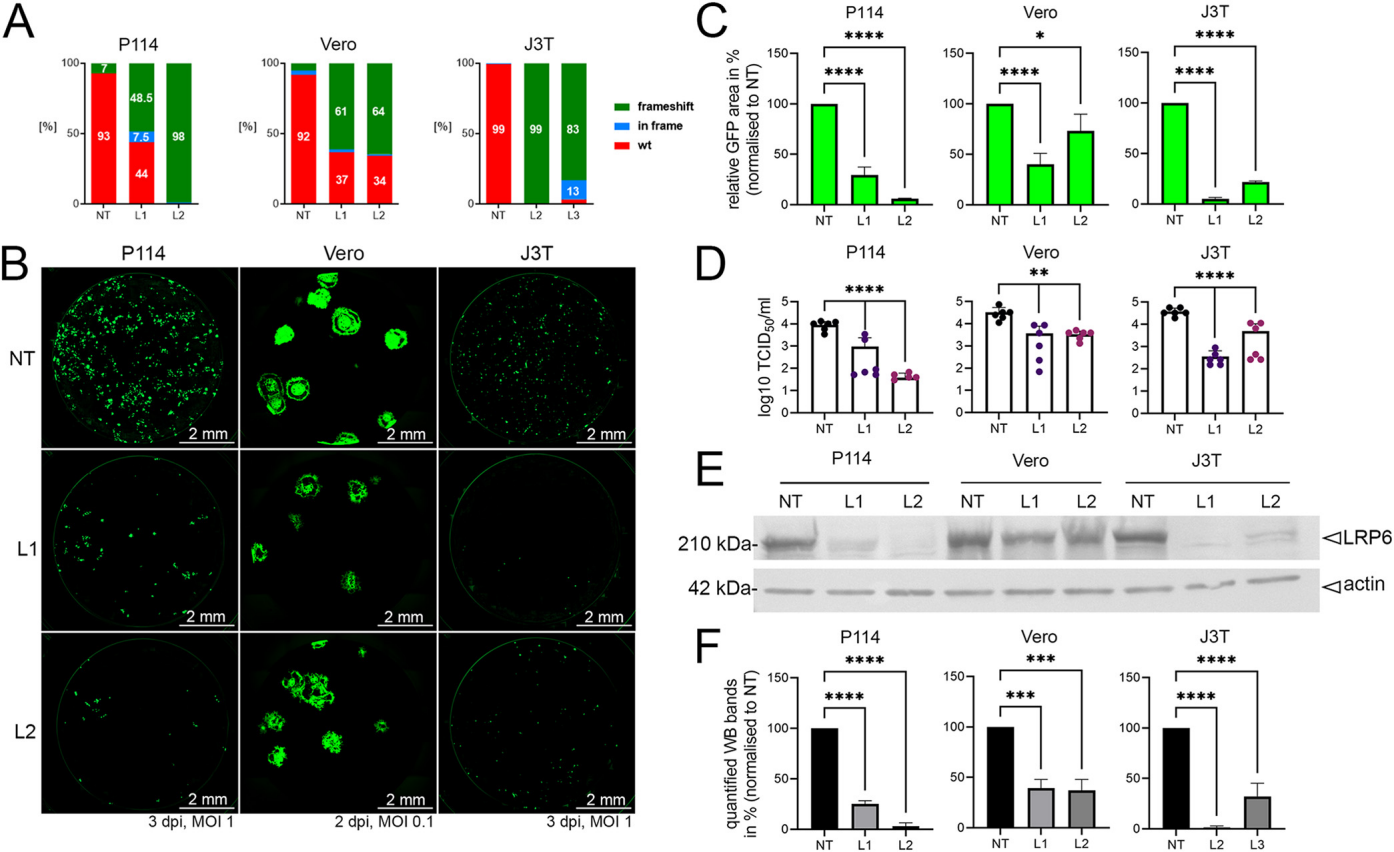
Ablation of LRP6 expression decreases CDV-OP infection in various canine cell lines. (A) Tracking of indels by decomposition (TIDE) analyses. Frequency of frameshift short insertions/deletions of LRP6^KO^ cells (green) using gRNA 1 or 2 (L1 and L2, respectively) or a nontargeting (NT) gRNA. In-frame mutations are color-coded in blue, and the wt genotype is color-coded in red. (B) Fluorescent syncytium formation of LRP6-expressing or ablated (LRP6^KO^) cells infected with OP^neon^. L1, LRP6^KO^ gRNA1; L2, LRP6^KO^ gRNA2; NT, nontargeting gRNA control. Pictures of stitched 96-wells were taken 3 days postinfection (dpi) (2 dpi for Vero cells). (C) Quantitative assessment of fluorescence emission from OP^neon^-infected cells, normalized to the NT. The means and standard deviations (SD) of data from three independent experiments are shown. Statistical significance was determined using a one-way ANOVA followed by Dunnett’s multiple-comparison test, using GraphPad Prism v.9 to analyze the differences between the values obtained from the NT and those from the indicated KO cells (*, *P* < 0.05; ****, *P* < 0.0001). (D) The viral titers of OP^neon^ in LRP6-expressing and LRP6^KO^ cells are displayed in Log_10_ TCID_50_/mL. The means and standard deviations (SD) of data from six independent experiments are shown. Statistical significance was determined using a one-way ANOVA followed by Dunnett’s multiple-comparison test, using GraphPad Prism v.9 to analyze the differences between the values obtained from the NT and those from the indicated KO cells (**, *P* < 0.01; ****, *P* < 0.0001). (E) Assessment of LRP6 expression in the indicated cells via Western blotting analyses, using an anti-LRP6 antibody. As a loading control, actin was detected using a monoclonal anti-actin antibody. (F) Band intensities were recorded with the Bio-1D software. The means and standard deviations (SD) of data from three independent experiments, normalized to the NT, are shown. Statistical significance was determined using a one-way ANOVA followed by Dunnett’s multiple-comparison test using GraphPad Prism v.9 to analyze differences between the NT and the LRP6^KO^ values (***, *P* < 0.001; ****, *P* < 0.0001).

To validate which factors are essential in promoting CDV-OP infectivity, we selected four genes encoding membrane proteins among our hits: *LRP6*, *STT3A*, *KNCE5*, and *ALCAM* (*LRP6*) was the most significant hit, with a false discovery rate (FDR) of <0.003 in both analyses performed (Table S3). For each gene, we generated single KO cells using two different sgRNA (1 and 2), whereas control cells were generated by inserting a nontargeting (NT) sgRNA. The KO efficacy was confirmed via a by tracking of indels by decomposition (TIDE) analysis (34) (Fig. 2A, Fig. S1B, and S2B). Remarkably, areas expressing neon (representing syncytium formation in infected cells) displayed a reduction of 94% in P114-LRP6^KO^ cells, exhibiting the highest frameshift mutation rate (P114-LRP6^KO/2^ or P114-L2), compared to NT controls. Similar results were obtained in other LRP6^KO^ cell lines, such as Vero and J3T Bg (J3T; a canine glioma cell line [35, 36]) (Fig. 2B and C). Corroborating these findings, the viral titers (determined by a 50% tissue culture infection dose [TCID_50_/mL]) obtained from the P114-L2 cells revealed a more than 2-log decrease, compared to titers determined from NT control cells (Fig. 2D). Of note, in all generated LRP6^KO^ cells, the reduction of OP^neon^ infection clearly correlated with the percentage of the determined frameshift mutation rate (Fig. 2A, C, and D) as well as the presence of proteins detected via Western blot (WB) (Fig. 2E and F). As a control, P114 cells (NT and L2) were infected with VSV particles that (i) carry the G glycoprotein-gene deleted genome as well as express additional reporters and (ii) are transcomplemented with G glycoprotein (VSVΔG/G) (37, 38). No reduction in L2-infected cells was monitored (Fig. S3A). In fact, VSVΔG/G replicated more efficiently in the P114-L2 cells, which revealed no impairment of the VSV replication machinery in the absence of LRP6.

10.1128/mbio.03114-22.3 FIG S3 **LRP6 specifically promotes CDV-OP infectivity.** (A) Infection assays in the indicated cells with VSVΔG/G in the presence or absence of the anti-VSV-G antibody. Since the VSVΔG genome also encodes the firefly luciferase reporter protein, viral infectivity was assessed indirectly by recording the firefly luciferase activity in infected cells 24 h postinfection. The means and standard deviations (SD) of data from three independent experiments are shown. Statistical significance was determined using a one-way ANOVA, followed by Dunnett’s multiple-comparison test, using GraphPad Prism v.9 to analyze the differences between the values obtained from the P114-NT cells and those from the indicated P114-LRP6^KO/2^ (L2) cells (***, *P* < 0.001; ****, *P* < 0.0001). (B) Assessment of the binding activity of various TST-tagged soluble H-protein constructs with membrane-anchored LRP6 proteins. The total cell lysates (TL) of the P114-L2/L-HA cells were mixed with the indicated soluble receptor-binding protein constructs. Whereas a mouse anti-TST antibody was used for the immunoprecipitation step, and immunoprecipitated (IP) and coimmunoprecipitated (coIP) proteins were revealed via Western blotting analyses that used a rat anti-HA or a mouse anti-TST antibody. (C) Infection assays in the indicated cells using VSVΔG complemented with its own glycoprotein (G) in the presence of the entry inhibitor 3G (an F-protein inhibitor). Since the VSVΔG genome also encodes the firefly luciferase reporter protein, viral infectivity was assessed indirectly by recording the firefly luciferase activity in infected cells 24 h postinfection. The means and standard deviations (SD) of data from three independent experiments are shown. Statistical significance was determined using a one-way ANOVA followed by Dunnett’s multiple-comparison test, using GraphPad Prism v.9 to analyze the differences between the values obtained from the indicated J3T cells and those from cells treated with the 3G inhibitor (ns, nonsignificant). Download FIG S3, TIF file, 2.5 MB. Copyright © 2023 Gradauskaite et al. 2023 Gradauskaite et al. https://creativecommons.org/licenses/by/4.0/ This content is distributed under the terms of the Creative Commons Attribution 4.0 International license.

10.1128/mbio.03114-22.6 TABLE S3 The false discovery rate (FDR) and log fold change (LFC) values obtained after an analysis (with MAGeCK or gscreend) of the CDV-treated group (D15) compared to D0 with two experiments. Download Table S3, DOCX file, 0.01 MB. Copyright © 2023 Gradauskaite et al. 2023 Gradauskaite et al. https://creativecommons.org/licenses/by/4.0/ This content is distributed under the terms of the Creative Commons Attribution 4.0 International license.

**FIG 3:**
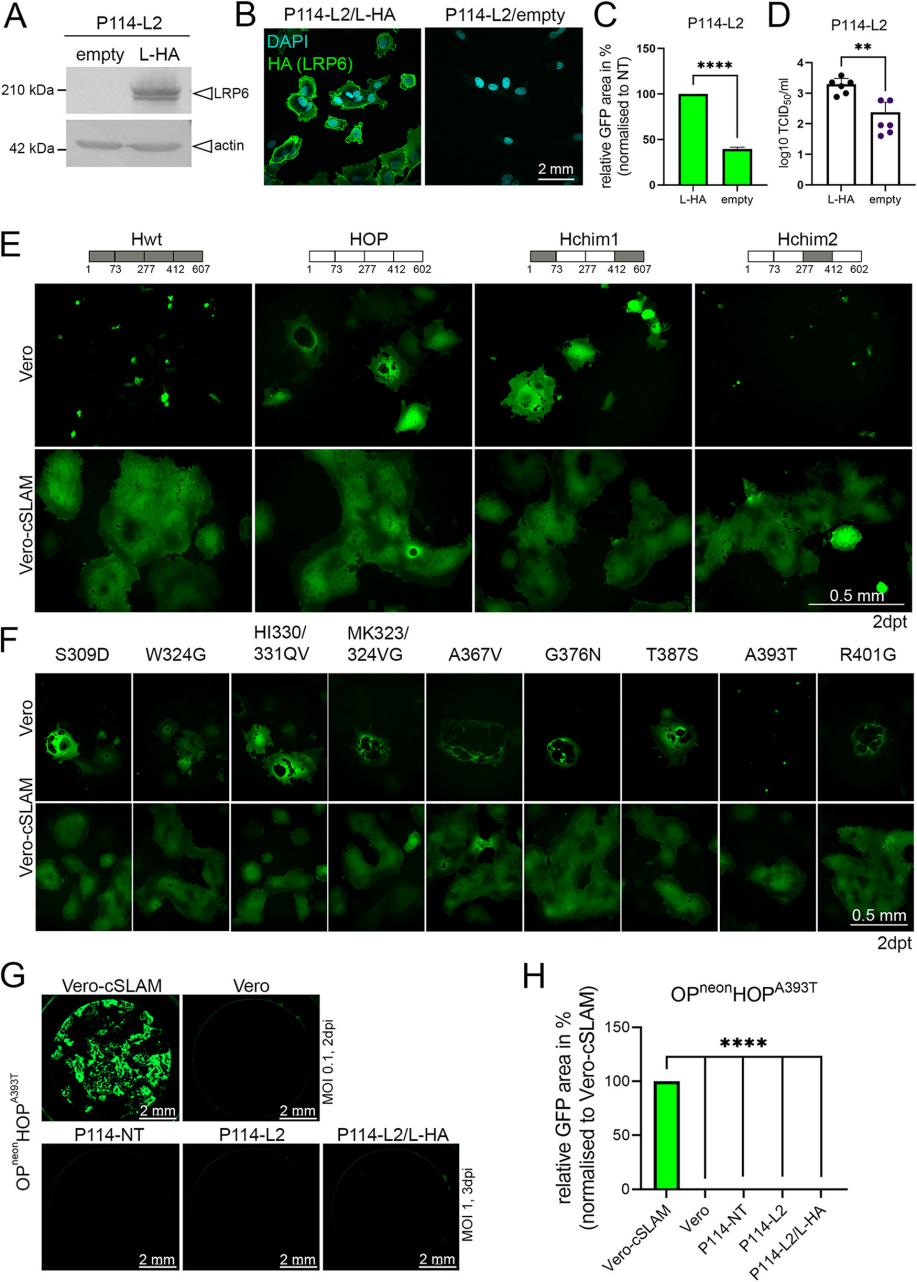
A point mutation in the CDV-OP receptor-binding protein (A393T) prevents infectivity in Vero and P114 cells. (A) Assessment of LRP6-HA protein expression via Western blotting analyses, using an anti-HA antibody in P114-LRP6^KO^ cells (P114-L2) reconstituted with cLRP6-HA (P114-L2/L-HA) or not (P114-L2/empty). (B) IF staining of indicated cells, using a monoclonal anti-HA MAb. (C) Quantitative assessment of fluorescence emission from OP^neon^-infected cells, using an MOI of 1. Images were taken at 3 dpi. The means and standard deviations (SD) of data from three independent experiments are shown. Statistical significance was determined using an unpaired *t* test, using GraphPad Prism v.9 to analyze the differences between the P114-L2/L-HA and P114-L2/empty values (****, *P* < 0.0001). (D) The viral titers of OP^neon^ in P114-L2/L-HA and P114-L2/empty cells are displayed in Log_10_ TCID_50_/mL. The means and standard deviations (SD) of data from six independent experiments are shown. Statistical significance was determined using an unpaired *t* test, using GraphPad Prism v.9 to analyze the differences between the values obtained from the indicated cells (**, *P* < 0.01). (E) Cell-cell fusion induced by cells transiently expressing the indicated H-proteins together with FOP and GFP. Schematic representation of the various H-proteins with the amino acid numberings of the exchanged H-fragments are shown. OP-derived fragments are colored-coded in white, and wt-derived fragments are highlighted in gray. Images were taken 2 days posttransfection (dpt). (F) Cell-cell fusion induced by cells transiently expressing the indicated H-protein variants harboring single (or double) point mutation(s) together with FOP and GFP. Images were taken at 2 dpt. (G) Infection of various cell lines with OP^neon^ carrying a single point mutation (A393T) in the H-protein (OP^neon^HOP^A393T^). Images were taken at 2 or 3 dpi for the Vero or other cell lines, respectively. (H) Quantitative assessment of the fluorescence emission from the OP^neon^HOP^A393T^-infected cells. The means and standard deviations (SD) of data from three independent experiments are shown. Statistical significance was determined using a one-way ANOVA followed by Dunnett’s multiple-comparison test, using GraphPad Prism v.9 to analyze the differences between the values obtained from the Vero-cSLAM cells and those from other cell types (****, *P* < 0.0001).

To exclude off-target effects on the OP^neon^ infectivity, we restored the expression of LRP6 in P114-L2 cells (P114-L2/L-HA). The stable expression of canine LRP6 harboring an N-terminal HA-tag was successfully obtained via the retroviral transduction of the P114-L2 cells, as demonstrated via immunofluorescence and Western blot analyses (Fig. 3A and B). The HA-tag allowed us to perform multiple selection cycles using the magnetic-activated cell sorting (MACS) technique to enrich the cells that were re-expressing LRP6. Control cells were generated via transduction with an empty vector (P114-L2/empty). Importantly, OP^neon^ infectivity significantly increased in the cells that were complemented with LRP6, compared to the control cells (Fig. 3C and D). Of note, while infectivity (measured in TCID_50_/mL) increased by about 1-log in the LRP6-complemented cells, the titers did not reach the levels observed in the control P114-NT cells (Fig. 2D, left panel). While such a discrepancy might have been caused by LRP6 overexpression (which might have interfered with cellular functions [e.g., LRP6-mediated signaling] and, in turn, impacted viral infection), we cannot exclude that the grafted HA-tag at the N-terminal region of LRP6 might have disturbed the viral infectivity. Nevertheless, although not fully restored, the infectivity significantly and reproducibly improved in the LRP6-complemented cells, compared to the LRP6^KO^ cells, which unambiguously demonstrated the role of LRP6 in promoting CDV-OP infection.

Interestingly, in STT3A-depleted P114 cells (P114-STT3A^KO/1^ and P114-STT3A^KO/2^ or P114-S1 and P114-S2), a significant reduction of infectivity was also observed, as measured by counting the GFP area (Fig. S1A and C, left panel) or by TCID_50_/mL (Fig. S1D, left panel). Similar trends were confirmed in Vero-STT3A^KO/1^ and Vero-STT3A^KO/2^ cells (Vero-S1 and Vero-S2) (Fig. S1A, C, and D, right panel). In contrast, the depletion of neither KNCE5 nor ALCAM significantly altered CDV-OP infectivity in P114 cells (Fig. S2A, C, and D). This is consistent with our screen results, in which *LRP6* and *STT3A* were the only significant hits in both analyses, with an FDR of <0.1 using the gscreend analysis (Table S3). *ALCAM* and *KCNE5* had an FDR of >0.5 in both analyses and were, therefore, less likely to validate.

10.1128/mbio.03114-22.1 FIG S1 **The ablation of the STT3A gene significantly reduces CDV-OP infections.** (A) Fluorescence emission from syncytia induced by OP^neon^ in STT3A-expressing or ablated (STT3A^KO^) P114 and Vero cells. Pictures were taken 3 dpi (2 dpi for Vero cells). S1, STT3A^KO^ gRNA1; S2, STT3A^KO^ gRNA2; NT, nontargeting gRNA control. (B) Tracking of indels by decomposition (TIDE) analyses. Frequency of frameshift short insertions/deletions of STT3A^KO^ cells (green) using gRNA 1 or 2 or a nontargeting (NT) gRNA. In-frame mutations are color-coded in blue, and the wt genotype is color-coded in red. (C) Quantitative assessment of fluorescence emissions from OP^neon^-infected cells. The means and standard deviations (SD) of data from three independent experiments are shown. Statistical significance was determined using a one-way ANOVA followed by Dunnett’s multiple-comparison test, using GraphPad Prism v.9 to analyze the differences between the values obtained from the NT cells and those obtained from the indicated STT3A^KO^ cells (****, *P* < 0.0001). (D) Viral titers of OP^neon^ in NT and STT3A^KO^ cells are displayed in Log10 TCID_50_/mL. The means and standard deviations (SD) of data from six independent experiments are shown. Statistical significance was determined using a one-way ANOVA followed by Dunnett’s multiple-comparison test, using GraphPad Prism v.9 to analyze the differences between the values obtained from the NT cells and those obtained from the indicated STT3A^KO^ cells (**, *P* < 0.01; ****, *P* < 0.0001). Download FIG S1, TIF file, 2.5 MB. Copyright © 2023 Gradauskaite et al. 2023 Gradauskaite et al. https://creativecommons.org/licenses/by/4.0/ This content is distributed under the terms of the Creative Commons Attribution 4.0 International license.

10.1128/mbio.03114-22.2 FIG S2 **Ablation of KNCE5 and ALCAM genes does not significantly reduce CDV-OP infections.** (A) Fluorescence emission from syncytia induced by OP^neon^ in KCNE5- and ALCAM-ablated (KCNE5/ALCAM^KO^) or expressing P114 cells. K1, KCNE5^KO^ gRNA1; K2, KCNE5^KO^ gRNA2; A1, ALCAM^KO^ gRNA1; A2, ALCAM^KO^ gRNA2; NT, non-targeting gRNA control. Pictures were taken 3 dpi using an MOI of 1. (B) Tracking of indels by decomposition (TIDE) analyses. Frequency of frameshift short insertions/deletions of KCNE5/ALCAM^KO^ cells (green) using gRNA 1 or 2 or a non-targeting (NT) gRNA. In-frame mutations are color-coded in blue, and the wt genotype is color-coded in red. (C) Quantitative assessment of fluorescence emissions from OP^neon^-infected KO and NT cells. The means and standard deviations (SD) of data from three independent experiments are shown. Statistical significance was determined using a one-way ANOVA followed by Dunnett’s multiple-comparison test, using GraphPad Prism v.9 to analyze the differences between the values obtained from the P114-NT cells and those from the indicated KCNE5/ALCAM^KO^ cells (ns, nonsignificant). (D) The viral titers of OP^neon^ in the NT and KCNE5/ALCAM^KO^ cells are displayed in Log10 TCID_50_/mL. The means and standard deviations (SD) of data from six independent experiments are shown. Statistical significance was determined using a one-way ANOVA followed by Dunnett's multiple-comparison test, using GraphPad Prism v.9 to analyze the differences between the values obtained from the NT cells and those from the indicated KCNE5/ALCAM^KO^ cells (ns, nonsignificant). Download FIG S2, TIF file, 2.5 MB. Copyright © 2023 Gradauskaite et al. 2023 Gradauskaite et al. https://creativecommons.org/licenses/by/4.0/ This content is distributed under the terms of the Creative Commons Attribution 4.0 International license.

Taken together, our data indicate that LRP6 and STT3A are two essential host factors that are required for CDV-OP infections. LRP6 is expressed at the cell surface, where it is known for its roles (i) in activating the Wnt/β-catenin signaling pathway, (ii) in simulating cell proliferation, and (iii) as an oncogene (39–41). In contrast, STT3A, a catalytic subunit of the N-oligosaccharyltransferase complex, functions in the endoplasmic reticulum to transfer glycan chains to asparagine residues of target proteins (42). Given these cellular localizations, we focused our investigations on LRP6 to analyze its role in CDV-OP infection.

### The HOP-A393T mutation results in a “blind” virus that loses infectivity in P114 and Vero cells.

To map the region of the receptor-binding protein (HOP) that promotes LRP6-dependent F-protein fusion triggering, we took advantage of the fact that the wild-type A75/17 CDV strain (wt) does not efficiently infect P114 cells. We constructed a series of chimeric receptor-binding (H) proteins, comprising different parts of the wt (Hwt) or OP (HOP) sequences (Fig. 3E). The H-protein variants were then expressed (together with the fusion protein [FOP] and Green Fluorescent Protein [GFP]) in Vero and Vero-cSLAM cells. Interestingly, chimera 2 (Hchim2) lost the fusion-promotion activity exclusively in Vero cells. Because Hchim2 harbors 11 amino acid (aa) differences in the region encompassing residues 277 to 412 between both H-protein sequences, we generated single or double (if two aa differences were successive) mutants and repeated the transient fusion assay. Clearly, only the HOP-A393T variant lost fusion-promotion in Vero cells, but it preserved this function in Vero-cSLAM cells (Fig. 3F). Importantly, similar phenotypes were confirmed in the context of a recombinant virus carrying this single amino acid substitution (OP^neon^HOP^A393T^) (Fig. 3G and H). The recombinant virus additionally lost infectivity in P114-L2, P114-NT, and P114-L2/L-HA cells, which suggests that the point mutation in HOP abrogates a crucial role of LRP6 in promoting CDV-OP infectivity. We note that the mutation added a potential N-glycosylation site in HOP (generating the 391-N/Q/T-393 motif).

### LRP6 acts as a receptor for cellular entry of CDV-OP.

**FIG 4:**
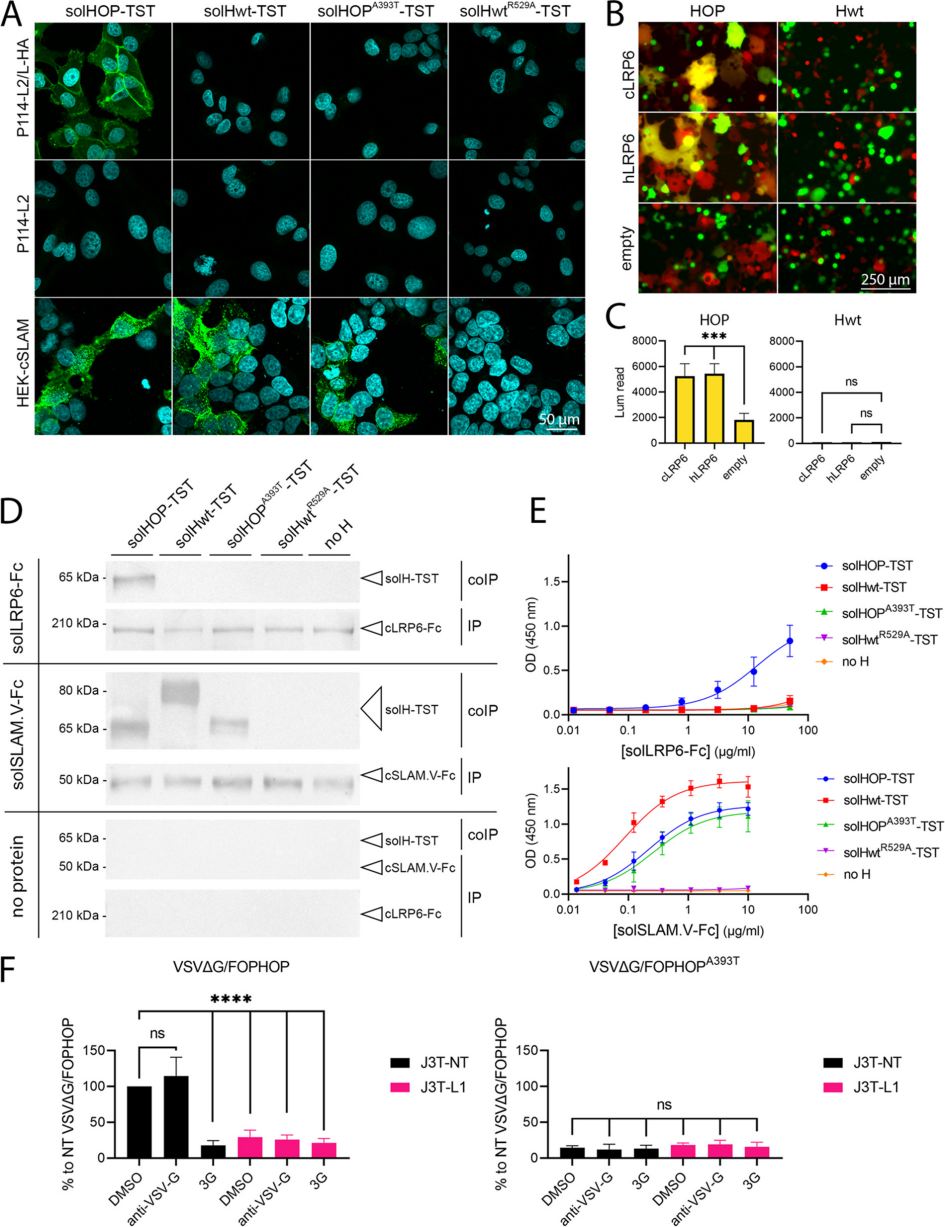
LRP6 acts as a functional receptor for CDV-OP. (A) Assessment of the binding activities of various solH-proteins at the cell surfaces of the indicated cells via immunofluorescence (IF) analyses, using an anti-TST monoclonal antibody. (B) Qualitative assessment of cell-cell fusion upon mixing two cell populations expressing the indicated H/F combinations, the human or canine LRP6 proteins (or empty vector control), the split nanoluciferase (nLuc) reporter proteins, and either GFP or RFP (see Materials and Methods for more details). Images of fluorescence emission were taken 24 h after cell mixing. (C) Quantitative assessment of the cell-to-cell fusion of mixed cell populations. Luminescence triggered (upon the addition of the substrate) by the reconstituted nLuc reporter protein, which was observed using a multiplate reader (Cytation 5 device, BioTek). The means and standard deviations (SD) of data from three independent experiments are shown. Statistical significance was determined using a one-way ANOVA followed by Dunnett’s multiple-comparison test, using GraphPad Prism v.9 to analyze the differences between the values obtained from the hLRP6- or cLRP6-transfected cells and those from the empty vector-transfected control cells. (***, *P* < 0.001; ns, nonsignificant). (D) Assessment of the binding activity of various TST-tagged, soluble H-protein constructs by mixing them with either an Fc-carrying soluble cLRP6 variant (solLRP6-Fc), an Fc-carrying soluble cSLAM protein (solSLAM.V-Fc), or without any proteins (for control experiments) via coimmunoprecipitation assays and Western blot analyses. Whereas protein G beads were used for the immunoprecipitation step, immunoprecipitated (IP) and coimmunoprecipitated (coIP) proteins were investigated via Western blotting analyses, using an anti-human-Fc or a mouse anti-TST antibody. (E) An ELISA of soluble proteins was used for the coIP experiments. (F) Infection assays in the indicated J3T cells were performed, using VSVΔG pseudotyped with standard CDV-OP glycoproteins (left) or a receptor-binding protein that was defective in LRP6-binding (HOP^A393T^; right). The assay was performed in the presence of an F-protein inhibitor (3G), an anti-VSV-G antibody, or DMSO. Since the VSVΔG genome also encodes the firefly luciferase reporter protein, the viral infectivity was assessed indirectly via the recording of the firefly luciferase activity in infected cells at 24 h postinfection. The means and standard deviations (SD) of data from three independent experiments are shown. Statistical significance was determined using a one-way ANOVA followed by Dunnett’s multiple-comparison test, using GraphPad Prism v.9 to analyze the differences between the values obtained from DMSO-treated J3T-NT cells and those from the indicated J3T cells that were treated with inhibitors or DMSO (****, *P* < 0.0001; ns, nonsignificant).

To further study whether LRP6 is the unknown receptor X of CDV-OP, we then determined the binding activity of the recombinantly expressed, soluble forms of receptor-binding proteins (solH) to the cell surfaces of P114 cells. For this set of experiments, we used the LRP6-HA expressing cells (P114-L2/L-HA) to facilitate the IF staining. Strikingly, when the P114-L2/L-HA cells were treated with solHOP carrying a TwinStrepTag (solHOP-TST), clear signals were detected at the cell surface, using an anti-TST monoclonal antibody (MAb), and this signal was absent in the LRP6^KO^ cells (P114-L2) (Fig. 4A, left panel). Furthermore, the treatment of P114-L2/L-HA with a soluble form of either the H-protein derived from the A75/17 CDV-wt strain (solHwt-TST), which does not efficiently infect P114 cells, or solHOP^A393T^-TST, did not show a positive signal (Fig. 4A, upper panel). In contrast, the solHOP-TST, solHOP^A393T^-TST, and solHwt-TST recombinant proteins did bind to HEK-293T cells that expressed the canine SLAM receptor (Fig. 4A, lower panel). To control the specificity of the SLAM binding activity in this experiment, we included a soluble protein carrying a point mutation that is known to disrupt the SLAM interaction (solHwt^R529A^-TST) (Fig. 4A, right panel) (10, 43, 44).

We then aimed to corroborate these findings via a quantitative cell-cell fusion assay, which solely relies on the proper bioactivity of both CDV glycoproteins and a cognate receptor. Briefly, while one cell population (effector cells) is transfected with plasmids expressing both glycoproteins (HOP and FOP) as well as one part of the nanoluciferase (nLuc) reporter protein (HiBiT fused to red fluorescent protein [RFP] via a P2A motif), the other cell population (target cells) is transfected with a plasmid encoding the receptor (LRP6) with the complementary part of nLuc (LgBiT fused to GFP via a P2A motif). Hence, if the H-protein binds to a cognate receptor when both of the cell populations are mixed, plasma membrane fusion is triggered, which thereby leads to cytosolic-content mixing. The latter event can be detected either by fluorescence microscopy (fused cells appear yellow by merging the GFP and RFP fluorescence channels) or by recording the light emission generated by the reconstituted nLuc reporter protein (23) (Fig. 4B). In this set of experiments, human LRP6 was also included. In sharp contrast, a significant 3-fold increase in luminescence emission was detected in human and canine LRP6-transfected cells that were mixed with HOP and FOP coexpressing cells (*P* ≤ 0.003) (Fig. 4C, left panel). As expected, the overexpression of the CDV glycoproteins of the wt strain did not trigger cell-cell fusion in HEK-293T cells (Fig. 4C, right panel). Of note, since we used HEK-293T cells, which naturally express low levels of human LRP6, the induction of small syncytia leading to a nLuc signal in the control cells that were transfected with the empty vector was expected.

As an additional piece of evidence that LRP6 is a supplementary CDV-OP receptor, we conducted coimmunoprecipitation (coIP) experiments. Here, LRP6-HA-expressing cells were lysed and subsequently treated with solHOP-TST, solHOP^A393T^-TST, solHwt-TST, or solHwt^R529A^-TST. All of the recombinant proteins were immunoprecipitated using the anti-TST Mab and were subjected to SDS-PAGE electrophoresis. Strikingly, as assessed via WB analyses, the LRP6 protein was efficiently coimmunoprecipitated with solHOP but not with solHwt, solHwt^R529A^-TST, or solHOP^A393T^-TST (Fig. S3B). To confirm the specific protein-protein interaction, we next engineered a soluble canine LRP6 protein harboring an Fc-tag (solLRP6-Fc; see Materials and Methods for more details) and repeated the coIP with the different soluble H-proteins together with a positive control (solSLAM.V-Fc) and a negative control (absence of Fc-carrying protein) (Fig. 4D). Our data clearly showed that only solHOP-TST interacts with solLRP6-Fc. In these experiments, solHOP-TST was somewhat less efficiently coimmunoprecipitated in the presence of solLRP6-Fc, compared to a solSLAM.V-Fc control construct. The direct interaction between LRP6 and HOP was validated using an enzyme-linked immunosorbent assay (ELISA), which showed a detectable interaction of LRP6 exclusively with solHOP (Fig. 4E, upper panel). Conversely, solHOP-TST, solHOP^A393T^-TST, and solHwt-TST, but not solHwt^R529A^-TST, potently interacted with solSLAM.V-Fc (Fig. 4E, lower panel). We confirmed that the binding efficiency of solHOP-TST with solLRP6-Fc was lower than that of solSLAM.V-Fc.

To further demonstrate that CDV-OP employs LRP6 as an essential entry factor, we made use of the pseudotyping VSV-based reporter system. This enables the efficient decoupling of the entry process, which relies on the investigated foreign viral glycoproteins from the replication step, which depends on the VSV replication complex. Therefore, we transiently expressed both of the CDV-OP glycoproteins (e.g., FOP and HOP) to complement the VSVΔG particles. The validation of proper glycoprotein integration was determined via the inoculation of the pseudotyped viruses on J3T-NT and -L1 cells. While the treatment of these cells with the fusion inhibitor 3G (45, 46) inhibited VSVΔG/FOPHOP infection, efficient infection was recorded in nontreated cells. No impact of anti-VSV-G neutralizing antibodies (nAb) was recorded (Fig. 4F, left panel). Importantly, VSVΔG/FOPHOP showed a significant reduction of infectivity in J3T-L1 (consisting of the highest percentage of cells with frameshift mutations), compared to the control NT cells (Fig. 4F, left panel). In contrast, the pseudotyped viruses harboring the HOP variant (VSVΔG/FOPHOP^A393T^) hardly infected the cells (Fig. 4F, right panel). Of note, the control VSVΔG/G particles efficiently entered and replicated in J3T-NT and -L1 cells (even in the presence of the CDV fusion protein inhibitor 3G), thereby indicating that there is no major impact of LRP6 on the VSV entry and replication stages (Fig. S3C).

Taken together, this series of diverse assays shows that LRP6 acts as a functional receptor for CDV-OP.

## DISCUSSION

The Onderstepoort vaccine strain of CDV (CDV-OP) not only is used as an efficient vaccine but also exhibits promising oncolytic activity (22, 47). Regarding the latter, CDV-OP has a natural tropism for different types of cancer cells (48, 49). Although wild-type CDV is known to bind to two host receptors (SLAM and N4), it is well-established that CDV-OP infects a wide variety of transformed cells that do not express those receptors (21, 22), including Vero, J3T-Bg, and P114 cells. To shed light on the host factors that are required for CDV-OP infection and to potentially decipher new mechanisms of cell entry, we performed a genome-wide CRISPR/Cas9 KO screen using the canine mammary carcinoma cell line P114, and we identified the surface-expressed LRP6 transmembrane protein as a top candidate. Whereas the genetic ablation of *LRP6* rendered cells significantly more resistant to CDV-OP infection, ectopic expression in KO cells conferred susceptibility, which validated the crucial role of this host factor on the regulation of the CDV-OP infectious cycle.

Several lines of evidence strongly suggest that LRP6 acts a functional entry receptor for CDV-OP: (i) the overexpression of LRP6 led to more efficient FOP/HOP-dependent cell-cell fusion, (ii) a soluble construct of LRP6 (solLRP6-Fc) stained the surface of LRP6-expressing cells, (iii) solHOP-TST was demonstrated to bind to the membrane-bound as well as to the soluble form of LRP6, (iv) a point mutation in HOP (A393T) that rendered CDV-OP noninfectious in P114, J3T, and Vero cells was demonstrated to interfere with the binding activity to LRP6, and (v) VSV particles pseudotyped with both CDV-OP glycoproteins exhibited significantly reduced infectivity profiles in LRP6^KO^ cells, compared to LRP6-expressing cells. Therefore, these data imply a direct functional interaction of the H protein of CDV-OP with LRP6 molecules that are expressed at the plasma membrane of a host cell.

Our data suggest that the affinity of the interaction between HOP and LRP6 is lower than that of SLAM. While the engineered soluble constructs of LRP6 and/or HOP might be responsible for the latter phenotype, we hypothesize that the affinity of the interaction might be limited. Although low affinity could affect CDV-OP infectivity, such impairments may be partially overcome by an overall increased avidity due to the concomitant binding of multiple H-proteins that are exposed on the viral particles to several LRP6 molecules. Future biochemical studies are required to unravel this phenotype.

We noticed that the point mutation in HOP prevented a detectable interaction with a soluble form of LRP6 and potentially added a supplementary N-glycosylation. Although it remains to be formally confirmed, our Western blot analyses indicate a slightly slower migration profile of solHOP^A393T^-TST in SDS-PAGE, compared to solHOP-TST (Fig. 4D), which would support the addition of an N-glycan at asparagine 391. Consequently, we hypothesize that this putative N-glycan may have interfered with the proper interaction with LRP6. Interestingly, the attachment protein (H) of the related Vero cell-adapted CDV-5804 strain also harbors a threonine at position 393 and is completely attenuated *in vivo*. Although this has to be validated in future experiments, this may indicate the possibility that CDV-5804 might have adapted to use another receptor to infect the Vero cells (50). Alternatively, the structures of the attachment proteins of related CDV strains may differ sufficiently such that they enable the binding to similar receptors via different modes.

Low-density lipoprotein (LDL) receptors belong to an evolutionarily ancient family of endocytic receptors that are known to be hijacked as docking sites by a group of viruses (51, 52), bacterial toxins (53, 54), and endogenous proteins that are associated with Alzheimer's disease (55, 56). Recently, Rift Valley fever virus (57) and Oropouche orthobunyavirus (58) were shown to bind LRP1, and the low-density lipoprotein receptor class A domain-containing 3 (LDLRAD3) was discovered to act as a receptor for Venezuelan equine encephalitis virus (59). Furthermore, LRP1 was also found to be an important host factor for the different stages of RNA virus infection, including VSV, Sandfly fever Sicilian virus, encephalomyocarditis virus, as well as Middle East respiratory syndrome coronavirus (MERS-CoV), severe acute respiratory syndrome coronavirus 1 (SARS-CoV-1), and SARS-CoV-2 (60).

LRP6 is a type I signal transmembrane protein that acts as a Wnt coreceptor for the canonical β-catenin signaling pathway, and it is known to be involved in the regulation of cell differentiation, proliferation, and migration (40). LRP6 is widely expressed in embryonic and adult tissues (61), and it is highly conserved between different species (62). Such expression profiles and high sequence identities fit nicely with the known broad cell tropism that is mediated by CDV-OP, which is known to infect various canine cells (22, 48) as well as SLAM- and N4-negative human Jurkat and African green monkey Vero cells (63, 64). LRP6 consists of a large ectodomain (EC), a transmembrane domain (TM), and a short cytosolic tail (65). The EC domain is composed of four globular YWTD β-propeller domains that are separated by EGF-like domains and low-density lipoprotein receptor (LDLR) type A repeats. Interestingly, while the TM segment is proposed to exhibit the inherent ability to form homo-oligomers (a mechanism suggested to lead to β-catenin activation [66]), the EC domain was recently found to assume different conformational states: (i) Wnt-activated, (ii) resting (ligand unbound), and (iii) Dkk1-inhibited (66). Thus, it would be interesting to investigate whether either intracellular LRP6 or the activated signaling pathway can regulate an additional stage of the viral infectious cycle (e.g., replication/transcription) in future experiments.

Moreover, LRP6 plays a crucial role in tumorigenesis, as increased LRP6 expression was found to trigger cell proliferation (41, 67) as well as metastasis (68, 69). Importantly, LRP6 is upregulated in around 60% of human malignant colorectal tissues (70) and breast cancers, especially in the subset of triple-negative tumors (71). Although LRP6 is expressed in normal tissue, attenuated CDV-OP could act as an attractive choice for oncolytic virotherapy, as cancer cell-specificity may also be controlled by defective interferon signaling, which is often found to be the case in cancer cells (72). Alternatively, the glycoproteins of CDV-OP may be employed to pseudotype attenuated MeV-Edm particles to potentially tackle the challenge of preexisting anti-MeV immunity. This may be of particular interest when the systemic delivery of oncolytic vectors is envisaged.

In summary, our data uncovered LRP6 as an essential entry mediator for the attenuated CDV-OP strain in various cell lines. Similar to CD46 for the attenuated strain of MeV, the discovery of LRP6 provides the grounds to shed light on the fundamental processes that are associated with viral-cell adaptation. Moreover, since LRP6 is upregulated in many cancer types *in vivo*, this offers solid bases upon which to further investigate the promising oncolytic properties of CDV-OP.

## MATERIALS AND METHODS

### Cell lines.

The canine mammary carcinoma cell line P114 (29) was kindly provided by Gerard R. Rutteman (University of Utrecht, Netherlands). The canine glioma cell line J3T-Bg (a J3T-derived cell line that was passaged in immunodeficient mice [Bg-Nude-SCID] and rederived to grow as an orthotopic tumor [35, 36]) was kindly provided by Dan York and Peter J. Dickinson (The University of California, USA). Vero cells stably expressing canine SLAM (Vero-cSLAM) were kindly provided by Yusuke Yanagi, Kyushu University, Japan). P114, J3T-Bg, Vero (ATCC CCL1-81), Vero-cN4 (45), Vero-cSLAM, BSRT-7 (73), HEK-293T/17 (HEK-293T; ATCC CRL-11268), and the HEK-293FT (RRID:CVCL_6911) cell lines were cultured in Dulbecco's Modified Eagle Medium (DMEM; Gibco) supplemented with 10% fetal calf serum (FCS, Sigma) and 50 units/mL penicillin-streptomycin (Gibco). Cell culture was carried out under standard conditions (37°C, 5% CO_2_).

### Plasmids.

The plasmid (pCMV6) encoding a C-terminally Myc-DDK-tagged human LRP6 protein (hLRP6) was ordered from Origene (NM_002336). The gene sequence encoding an N-terminally HA-tagged canine LRP6 protein (cLRP6) (UniProt: A0A8C0NS55_CANLF) was codon optimized (for mammalian cell expression systems) and synthetized at Twist Bioscience. The cLRP6 gene was cloned (without the HA tag) into the pCMV6 expression vector (In-Fusion; TaKaRa Bio). Empty pCMV6 vector was generated by deleting the hLRP6 gene. Expression plasmids encoding the glycoproteins of the Onderstepoort CDV strain (pCI-HOP and pCI-FOP), the glycoproteins of the A75/17 wild-type CDV strain, and the plasmid expressing the canine SLAM protein (pCI-cSLAM), were previously described (74, 75). Expression plasmids encoding the split nanoluciferase fragments RFP-HiBiT and GFP-LgBiT (76) were subcloned into the pCI expression vector (In-Fusion, TaKaRa Bio). cLRP6-HA was additionally cloned into the pOZ-N-FH vector (kindly provided by Dipanjan Chowdhury, Harvard Medical School). To engineer Hchim1, an NruI restriction site was first created (gene sequence 210 to 216) in the Hwt gene (without changing the aa sequence) (In-Fusion, TaKaRa Bio). Then, a gene fragment of HOP (encompassing amino acids [aa] 73 to 412) was amplified via PCR (TaKaRa Bio) and cloned into the NruI/XbaI-digested pCI-Hwt expression vector (In-Fusion Kit; TaKaRa Bio). Hchim2 was synthetized by Twist Bioscience. HChim2 harbors most of the regions of HOP with a fragment of Hwt inserted (gene sequence encompassing aa 277 to 412 of Hwt). Point mutations were performed in the HOP backbone (In-Fusion Kit; TaKaRa Bio). All primers are available upon request.

### Soluble proteins.

A dimeric form of soluble Hwt (solH-TST) as well as soluble V-domain canine SLAM (solSLAM.V-Fc) were previously described (43). The gene encoding a soluble form of the canine LRP6 protein (UniProt: A0A8C0NS55_CANLF: aa 20 to 1,246) fused to a human Fc domain (solLRP6-Fc) was synthesized at Twist Bioscience. TST-tagged proteins were produced and purified as described previously (77). Briefly, plasmids (3 mg) were sent to the Protein Production and Structure Core Facility of the EPFL (Switzerland) for expression (7 days in ExpiCHO cells). Subsequently, the protein was purified from 1 L of supernatant using a 5 mL StrepTrapXT column (Cytavia) and eluted with 500 mM biotin (Cytiva). Fc-tagged proteins were purified using HiTrap rProtein A FF (Cytiva).

### Reverse transcription PCR.

Cells were seeded in 6-well plates (3 × 10^5^ cells/well). The next day, the cells were trypsinized and centrifuged for 5 min at 300 g. The supernatant was discarded, and the pellet was resuspended in 350 μL RLT buffer (Qiagen) with 1% BME (M6250, Sigma-Aldrich). The samples were then homogenized using QIAshredder (Qiagen), and the total RNA was purified with an RNeasy Minikit (Qiagen), following the manufacturer’s instructions. cDNA was then produced using GoScript Reverse Transcriptase (Promega), following the manufacturer’s instructions. Target loci (using the primers listed in Table 1) were amplified using CloneAmp HiFi PCR Premix (TaKaRa), following these steps: (i) 98°C for 30 s; (ii) 30 cycles at 95°C for 10 s, 55°C for 5 s, and 72°C for 35 s; and (iii) 72°C for 2 min. The PCR product was then stained with TriTrack DNA Loading Dye 6× (Thermo Fisher Scientific), and electrophoresis was performed using 1% agarose gel. Fluorescent images were taken with a Quantum imaging device, using the VisionCapt software (Vilber Lourmat).

**TABLE 1:** Oligonucleotides used for RT-PCR

Gene	Primer	Sequence (5′–3′)
GAPDH	Forward	GCCTCCTGCACCACCAAC
	Reverse	CATACCAGGAAATGAGCT TG
N4	Forward	ACAGCAGAGGGCAGCCC
	Reverse	TTCTGGGTCATCTGCTG

### Lentiviral transduction.

Lentiviruses were generated via transient-transfection in HEK-293FT cells. The HEK-293FT cells were cultured in 150 cm^2^ dishes (Sigma) and grown to 70% confluence. The next day, the cells were transiently transfected with lentiviral packaging plasmids (ENV, pMDLg, REV) and the plentiCRISPRv2 vector containing the library plasmid or respective sgRNA using 2× HBS (280 nM NaCl, 100 mM HEPES, 1.5 mM Na_2_HPO_4_, pH 7.22), 2.5 M CaCl_2_, and 0.1× TE buffer (10 mM Tris [pH 8.0], 1 mM EDTA [pH 8.0], diluted 1:10 with dH_2_O). After 16 h, the medium was replaced with DMEM (Gibco) supplemented with 10% fetal calf serum (FCS, Sigma) and 50 units/mL penicillin-streptomycin (Gibco). After 30 h, the supernatant was harvested, passed through a 0.45 μm filter (ThermoFisher), and centrifuged at 20,000 × *g* for 2 h at 4°C in a SW40 rotor. After the supernatant was carefully discarded, the pellet was resuspended in 500 μL of phosphate-buffered saline (PBS) and stored at −80°C. Viral titers were determined using a qPCR Lentivirus Titration Kit (Applied Biological Materials).

For the lentiviral transduction, 1.5 × 10^5^ cells were seeded in 6-well plates. 24 h later, viruses (MOI of 30) were added on the cells together with 8 mg/mL polybrene (Merck Millipore). 24 h later, the virus-containing medium was replaced with medium containing puromycin (3.5 mg/mL, Gibco). Puromycin selection was performed for 5 days. The target site modifications of the polyclonal cell pools were monitored via a TIDE analysis (see below).

### Genome-wide CRISPR screen.

Based on the canine CanFam3.1 assembly, we established a genome-wide, lentivirus-based CRISPR/Cas9 library with more than 80,000 sgRNAs, targeting 20,039 protein-coding genes (4 sgRNAs per gene) in addition to 500 nontargeting and 500 intergenic controls (Table S1). The library was cloned into the lentiCRISPRv2 (pXPR_023) vector (Cas9+guide vector) at the Broad Institute (MA, USA).

P114 cells (4 × 10^8^) were seeded in 30 T175 cell culture flasks (Corning). After 24 h, lentivirus was added at an MOI of 1 with 8 mg/mL of polybrene (Merck Millipore). The virus-containing medium was replaced with medium containing puromycin (3.5 mg/mL, Gibco) at 24 h postransduction. Puromycin selection was performed for 3 days. Then, the cells were harvested and either pelleted (2.5 × 10^9^; labeled as D0) or seeded (2.5 × 10^9^ cells per condition, allowing a coverage of 300 cells per sgRNA). 14 T175 cell culture flasks (Corning) were used per experiment. Two independent experiments were performed. The control cells were split at the same time as the treated (infected) cells. Briefly, upon the infection of P114 cells with OP^neon^ at an MOI of 10, the cells were incubated for 2 h at 37°C on a rocking platform, and the medium was replaced with DMEM supplemented with 10% FCS. Infected cells were maintained in culture for 3 to 5 days until the surviving/resistant cells reached confluence. Then, the cells were harvested and reseeded for the next round of infection (four successive rounds of infection were performed). At day 15, the cells were pelleted, and genomic DNA (gDNA) was isolated using a QIAamp DNA Blood Midi Kit (Qiagen) and amplified using PCR P5 stagger primer mix (100 μM, AATGATACGGCGACCACCGAGATCTACACTCTTTCCCTACACGACGCTCTTCCGATCT [s] TTGTGGAAAGGACGAAAC*A*C*C*G, where [s] is the barcode region) and the uniquely barcoded P7 primer (5 μM, CAAGCAGAAGACGGCATACGAGAT [s] GTGACTGGAGTTCAGACGTGTGCTCTTCCGATCTCCAATTCCCACTCCTTTCAAG*A*C*C*T, where [s] is the barcode region). The PCR cycling conditions were as follows: (i) 95°C for 1 min, (ii) 94°C for 30 s, (iii) 52.5°C for 30 s, (iv) 72°C for 30 s, (v) go to step ii, × 27, and (vi) 72°C for 10 min. The PCR products were purified with Agencourt AMPure XP SPRI beads, according to the manufacturer’s instructions (Beckman Coulter, A63880). The samples were sequenced on a HiSeq2500 High Output (Illumina) with a 5% spike-in of PhiX. The statistical analysis was performed using MAGeCK software (31) and the gscreend R package (33).

### Genome editing.

The CRISPR/Cas9 plasmids were generated using a modified version of the lentiCRISPR v2backbone (RRID: Addgene_52961) in which a puromycin resistance ORF was cloned. sgRNA sequences were cloned in the lentiCRISPR v2 backbone using custom DNA oligonucleotides (Microsynth) that were melted at 95°C for 5 min, annealed at room temperature for 2 h, and subsequently ligated with quick-ligase (NEB) into a BsmBI-digested (Fermantas) backbone. All of the construct sequences were verified via Sanger sequencing. sgRNA sequences were chosen from the customized CRISPR dog library (for the canine constructs) or the Brunello library (for the primate constructs, Addgene_73178) (Table 2). Note that for the J3T-Bg cells, we named sgRNA2 as L1 and sgRNA3 as L2.

**TABLE 2:** Oligonucleotides used for genome editing

Gene	Species	Guide	Sequence (5′–3′)	Exon targeted
LRP6	Canis lupus familiaris	sgRNA1	GCCACCATTATCAATTCCACA	5
		sgRNA2	GCAAGGCTCAATGGTACCATG	6
		sgRNA3	GCCCGGACATTCCGAAGACTG	13
	Chlorocebus sabaeus	sgRNA1	GTTGCCTTAGATCCTTCAAGT	2
		sgRNA2	GCAAGGCTCAATGGGACCATG	6
STT3A	Canis lupus familiaris	sgRNA1	GACAGATCGAGAGATATACCC	6
		sgRNA2	GCTGCGCAGATAATCCACAA	9
	Chlorocebus sabaeus	sgRNA1	GACAGACATTCCGAATGTCGA	5
		sgRNA2	GCTGCGCAGGTAATCCACAA	9
KCNE5	Canis lupus familiaris	sgRNA1	GCCGCAATCTCGTCGACGTCA	1
		sgRNA2	GCTGCTTGAGCTGCACCATCG	1
ALCAM		sgRNA1	GATCCAGACGGCAACATCACA	5
		sgRNA2	GATTTGTCCTTAAACCCAAG	9
Non targeting gRNA (NT)			GTGATTGGGGGTCGTTCGCCA	No gene targeted

### gDNA isolation, amplification, and tracking of indels by decomposition (TIDE) analysis.

To evaluate the modification rate, genomic DNA was extracted from cell pellets using a QIAmp DNA Minikit (Qiagen), according to the manufacturer’s protocol. A 3-step protocol using Phusion High Fidelity Polymerase (Thermo Fisher Scientific) was followed to the amplify target loci: (i) 98°C for 30 s; (ii) 30 cycles at 95°C for 10 s, the temperature of melting (*T_m_*)°C for 20 s, and 72°C for 20 s; and (iii) 72°C for 5 min. PCR products were purified using a QIAquick PCR Purification Kit (Qiagen), according to the manufacturer’s protocol. Sanger sequencing confirmed the target modifications using the TIDE algorithm (34). The primers used in this PCR are listed in Table 3, as are the different *T_m_*°C values for each primer pair.

**TABLE 3:** PCR primers used for TIDE analysis

Gene	Species	Guide	Primer	Sequence (5´-3´)	*T_m_* (°C) for PCR protocol
LRP6	Canis lupus familiaris	sgRNA1	Forward	AGGACCTGGATGACATTTGGT	63°C
			Reverse	GCCCTAAAACACAAAACACCC	
		sgRNA2	Forward	AGCACGAAGGACAGACTTGA	
			Reverse	GCCACTGAAGAGCTGTTTACT	
		sgRNA3	Forward	TGAGGAGAGTTTCTGGGGAC	
			Reverse	AGGGTGGTTGGTTACTGTAATT	
	Chlorocebus sabaeus	sgRNA1	Forward	TGTAGTTGGAGGCTTGGAGG	64°C
			Reverse	AGGGTGGTGTATGTCAGTGG	
		sgRNA2	Forward	AGGTGCCACAGAATTATTGCT	63°C
			Reverse	AGAGCTTCTTACCCAACCATG	
STT3A	Canis lupus familiaris	sgRNA1	Forward	AGTCTTGGTATCATCATGGGGA	63°C
			Reverse	TGGAGGAGATGACAGAGAAAGA	
		sgRNA2	Forward	TTGGCCCTAATTCCTCGGAG	64°C
			Reverse	CTGGGCTGACAAGTGGACTA	
	Chlorocebus sabaeus	sgRNA1	Forward	AGAGTGGAGATGGGTGGTTT	64°C
			Reverse	CCACCCTCCAGCATCGATTA	
		sgRNA2	Forward	ACCTGAGCAAAAGCCAGAGA	64°C
			Reverse	GAGCCTTCCCTACCTGTCAG	
KCNE5	Canis lupus familiaris	sgRNA1	Forward	GGCTACGCACTCTCCTCAAC	64°C
			Reverse	CTGGAAGGGGAGAGAAGGAG	
		sgRNA2	Forward	CTCTGTCTCTCTCTCGCCTG	
			Reverse	AGGCAGGCGTAGAAGATCAT	
ALCAM		sgRNA1	Forward	GTCCACAGTCAATGCTTGGG	64°C
			Reverse	CTGCTCTTGATTCCTCTGCAC	
		sgRNA2	Forward	TGACCATTTCCTTGTCCAAACT	63°C
			Reverse	AAAGAGAGCAGAGGTGTGGG	

### Production of recombinant OP^neon^.

Recombinant OP^neon^ was generated by inserting the mNeonGreen sequence as a separate transcription unit before the N gene. Then, the reverse genetic system for CDV-OP (28) was used to rescue the recombinant virus. Briefly, the full-length, CDV-OP-expressing plasmid was cotransfected in BSR-T7 cells in a 6-well plate (Corning) (TransIT-LT1, Mirus Bio), together with pTM-N, -P/Cko, and -L expressing plasmids (3, 0.3, 0.3, and 0.1 μg, respectively). The next day, the plates were incubated for 2 h at 42°C, and the medium was exchanged. The BSR-T7 cells were then cocultured with Vero-cSLAM cells. After 3 days, the viruses were harvested and further amplified by infecting Vero cells in a T75 flask (Corning). When the syncytia reached about 50%, cell-associated and cell-free viruses were harvested (two cycles of freeze/thaw), centrifuged at 500 × *g* for 5 min at 4°C, transferred to new tubes, and frozen at −80°C. The TCID_50_/mL of the samples was determined in Vero-cSLAM cells using the Spearman and Kärber method.

### Infection assays.

Cells were seeded in a 96-well plate at 80% confluence (1 to 3.5 × 10^4^ cells/well). The cells were infected with OP^neon^ at MOI of 1 (MOI of 0.1 for Vero cells), and at 3 days postinfection (2 dpi for Vero cells), images of GFP fluorescence were taken using a Cytation 5 cell imaging multimode reader with a 4× objective. The images were stitched and analyzed using Gen5.10 software to measure the sum of the area of the GFP pixels (excluding the autofluorescent signals of well borders).

### Western blot analyses.

Cells were lysed in 0.5 mL of Laemmli buffer (2×) (Sigma-Aldrich) that contained 200 mM dithiothreitol (DTT, Thermo Fisher Scientific). The cell lysates were then homogenized via sonication for 15 s (Branson 450 Probe Sonifier), boiled at 95°C for 10 min, centrifuged at 16,000 × *g* for 2 min, and loaded onto Novex WedgeWell 4 to 20% Tris-Glycine gels (Invitrogen). Western blot analyses were performed using either rabbit monoclonal anti-LRP6 (C5C7, rabbit MAb, cell signaling, 1:100, overnight), mouse monoclonal anti-actin (A2066, Sigma-Aldrich, 1:3000, overnight), or anti-HA high-affinity rat MAb (3F10, Roche; 1:2000, overnight). A horseradish peroxidase (HRP)-conjugated goat anti-rabbit (P0448, Dako, 1:3000, 2 h), goat anti-mouse (P0447, Dako, 1:3000, 2 h), or goat anti-rat (Abcam; ab97057; 1:3000, 2 h) was used as the secondary antibody. Signals were detected on nitrocellulose membranes (Thermo Fisher Scientific) using a chemical colorimetric substrate (1-Step Ultra TMB-Blotting Solution, Thermo Fisher Scientific), following the manufacturer’s instructions. Pictures were taken with a Quantum imaging system (Vilber Lourmat) and quantified using the built-in Bio-1D analysis software.

### Coimmunoprecipitation.

**(i) *Membrane-anchored LRP6.*** P114-L2/L-HA cells were seeded in 15 cm tissue culture dishes. The next day, cells were washed with cold PBS and lysed in RIPA buffer containing protease inhibitor (cOmplete mix, Roche). The cell lysates were transferred into tubes, passed through a 26 G size needle (0.45 × 25 mm BL/LB, B Braun), and centrifuged at 4°C at 3,200 × *g* for 20 min. The supernatants were transferred into new tubes. In separate tubes, anti-TST Ab (THE NWSHPQFEK-tagged mouse MAb, A01732, GenScript, 1:150) was mixed with 20 μL of magnetic beads (Dynabeads Protein G, Invitrogen) in 300 μL of PBS containing 0.05% Tween 40 (PBS-T, Merck) and was incubated at 4°C for 10 min. Next, 15 μg of soluble HOP-TST, Hwt, HOP^A393T^-TST, and Hwt^R529A^-TST proteins were added to the Dynabeads/Ab mixture and incubated for 30 min on a rotating wheel at 4°C. The H protein/Ab/Dynabeads complexes were then mixed with the harvested supernatants and incubated on a rotating wheel overnight at 4°C. After five washes with PBS containing 0.05% Tween 40 (PBS-T, Merck), the bead samples were suspended in 40 μL of Laemmli buffer (2×) supplemented with 200 mM DTT and boiled at 95°C for 10 min. The samples were subjected to Western blot analyses using 4 to 8% Tris-Acetate gel (Invitrogen) either by using an anti-HA high-affinity rat MAb (3F10, Roche; 1:2000, overnight) followed by incubation with a HRP-conjugated goat anti-rat antibody (Abcam; ab97057; 1:3000, 2 h) or by using an anti-TST Ab (THE NWSHPQFEK-tagged mouse MAb, A01732, GenScript; 1:2000, overnight) followed by a HRP-conjugated polyclonal goat anti-mouse Ab (P0447, Dako; 1:3000, 2 h).

**(ii) *Soluble LRP6.*** 2 μg of purified solLRP6-Fc or solSLAM.V-Fc were mixed with 2 μL of magnetic beads (Dynabeads Protein G, Invitrogen) in 300 μL of PBS containing 0.05% Tween 40 (PBS-T, Merck) using Protein LoBind tubes (L200956G, Eppendorf) and incubated on a rotating wheel overnight at 4°C. Then, 0.5 μg of soluble HOP-TST, Hwt, HOP^A393T^-TST, and Hwt^R529A^-TST (or no proteins for the control experiments) were added to each bead sample and incubated on a rotating wheel for 2 h at 4°C. After five washes with PBS containing 0.05% Tween 40 (PBS-T, Merck), the bead samples were resuspended in 10 μL of Laemmli SDS reducing (4×) sample buffer (J60015, Alfa Aesar) supplemented with 200 mM DTT and were boiled at 95°C for 10 min. Finally, the samples were subjected to Western blot analyses (using 10% Bis-Tris gels [GenScript]), as described above for the membrane-anchored LRP6 coimmunoprecipitation. A HRP-conjugated, goat anti-human IgG antibody (AP113P, Merckmillipore, 1:2000, overnight) was used to reveal the Fc proteins.

### MACS cell sorting.

Cells (10 × 10^7^) were pelleted and resuspended in 500 μL of PBS supplemented with 0.5% BSA (Bovine Serum Albumin Fraction V, Roche) and 2 mM EDTA. An anti-LRP6 antibody (C10, Santa Cruz Biotechnology) was added to the cell suspension and incubated on a rotating wheel at 4°C for 1 h. The cells were then enriched using anti-mouse IgG Microbeads (Miltenyi Biotech) and LS columns (Miltenyi Biotec), according to the manufacturer's instructions. In order to further increase the percentage of LRP6-expressing cells, similar procedures were repeated twice.

### Qualitative fusion assay.

Vero and Vero-cSLAM cells were seeded in 24-well plates (Corning) (30,000 cells/well). The next day, the cells were transfected with plasmids encoding various H protein constructs together with FOP and green fluorescent protein (GFP) (0.4, 0.8, and 0.3 μg, respectively) by using TransIT-LT1 (Mirus Bio). Images of GFP fluorescence were taken 2 days posttransfection (dpt) with an EVOS 5000 fluorescence microscope, using the 10× objective.

### Quantitative fusion assay.

The quantitative fusion assay was performed as previously described (78) with minor modifications. In a 96-well plate (polystyrene microplates with a microclear bottom; Greiner Bio One), effector HEK-293T cells (2.5 × 10^4^/well) were transfected (TransIT-LT1, Mirus Bio) with plasmids encoding HOP, FOP, and RFP-HiBiT or Hwt, Fwt, and RFP-HiBiT (0.066 or 0.133, 0.1 μg per well, respectively). Simultaneously, in a 24-well plate, target HEK-293T cells (1.25 × 10^5^/well) were transfected with plasmids encoding the human LRP6 protein, canine LRP6 protein or empty vector control together with GFP-LgBiT (0.5 μg per well). 24 hours posttransfection, the target cells were trypsinized and equally divided into 4 wells of effector cells (generating quadruplicates). 3 hours later, the cocultured cells were treated with 40 μL Opti-MEM (Thermo Fisher Scientific) containing 0.2 μL of nanoluciferase substrate diluted in buffer solution (Nano-Glow Luciferase Assay System, Promega). The luminescence intensities were measured using a Cytation 5 microplate reader (BioTek), and images of the fluorescence emissions were captured using an EVOS M5000 fluorescence microscope (Thermo Fisher Scientific).

### Immunofluorescence analysis.

**(i) *Soluble H protein binding activity.*** A 24-well plate (Corning) was seeded with HEK-293T, P114-L2, or P114-L2/L-HA cell. Cells, transfected (or not for the P114 cells) with a plasmid encoding the HA-tagged cSLAM receptor were fixed at 24 h posttransfection (HEK-293T cells) or at 24 h postseeding (P114 cells) with 4% paraformaldehyde (PFA) and blocked with 1% milk solution in PBS containing 0.05% Tween 20 (PBS-T, Merck) for 2 h. The cells were then treated with 5 μg of soluble TST-tagged H proteins (solHOP-TST, solHwt, solHOP^A393T^-TST, or solHwt^R529A^-TST) and incubated for 1 h at room temperature (RT). Analyses were performed using either a DY-488-conjugated mouse monoclonal anti-Strep-Tactin XT Ab (2-1562-050, IBA; 1:1000, 1 h) (for the P114 cells) or THE NWSHPQFEK-tagged Mouse MAb (GenScript, A01732, 1:1000, 1 h) (for the cSLAM transfected HEK-293T cells). For the latter, Alexa Fluor 488-conjugated goat anti-mouse IgG (H+L) (A-11001, Invitrogen; 1:1000, 1 h) was used as a secondary antibody. The nuclei were stained with DAPI (62248, Thermo Fisher Scientific, 1:1000, 1 h). Pictures were acquired via laser scanning confocal microscopy (Olympus FV3000) and were then analyzed and composed using Fiji software.

**(ii) *Nectin-4 and LRP6 surface expression*.** A 24-well plate (Corning) was seeded with P114-L2, P114-L2/L-HA, Vero, or Vero-cN4. At 24 hours postseeding, the cells were treated with 4% PFA. To detect Nectin-4 (N4), cross-reacting anti-human N4 Abs (AF2659, R&D Systems, 1 h) (or goat serum [X0907, Dako] as a control) were used at concentrations of 5 μg/mL. This was followed by the Alexa Fluor 488-conjugated donkey anti-Goat IgG (H+L) cross-adsorbed secondary antibody (A-11055, Invitrogen, 1:1000, 1 h) together with DAPI (62248, Thermo Fisher Scientific, 1:1000, 1 h). To detect LRP6-HA, the cells were stained with anti-HA high-affinity rat IgG1 (clone 3F10, Roche, 1:1000, 1 h). This was followed by the Alexa Fluor 488-conjugated goat anti-Rat IgG (H+L) cross-adsorbed secondary antibody (A-11006, Invitrogen, 1:1000, 1 h) together with DAPI (62248, Thermo Fisher Scientific, 1:1000, 1 h). Fluorescent images were taken as described above.

### CDV pseudovirus neutralization.

Pseudovirus neutralization assays have been previously described (79, 80). Briefly, propagation-defective, CDV-OP glycoproteins-pseudotyped vesicular stomatitis virus (VSV) was produced by cotransfecting HEK-239T cells with HOP and FOP or with HOP^A393T^ and FOP, respectively. The cells were further inoculated with a glycoprotein G transcomplemented VSV vector (VSV*ΔG[Luc]) encoding enhanced green fluorescence protein (eGFP) and firefly luciferase reporter genes but lacking the glycoprotein G gene (38). After 1 h of incubation at 37°C, the inoculum was removed, and the cells were washed once with medium and were subsequently incubated for 24 h in medium containing 1:2000 of an anti-VSV-G MAb I1 (ATCC, CRL2700TM). Pseudotyped particles were then harvested and cleared via centrifugation. For the CDV pseudotype neutralization experiments, pseudovirus was incubated for 1 h at 37°C either with or without 3G (20 μM) or MAb I1. Subsequently, H protein- and F protein-pseudotyped VSV*ΔG(Luc) were added to J3T-NT and J3T-L1 cells that were grown in 96-well plates (25,000 cells/well). At 24 h postinfection, the luminescence (firefly luciferase activity) was measured using a ONE-Glo Luciferase Assay System (Promega) and a Cytation 5 cell imaging multimode reader (BioTek).

### ELISA.

First, 2 μg/mL of soluble dimeric H (HOP-TST, Hwt-TST, HOP^A393T^-TST, Hwt^R529A^-TST, or PBS [absence of H proteins]) were coated onto 96-well ELISA microplates (Corning) in duplicates and were left overnight at 4°C. The next day, the wells were washed 3 times with PBS-T and blocked with a 5% skimmed milk solution in PBS-T for 2 h. Then, serial 1/4 dilutions of soluble canine SLAM.V-Fc or canine LRP6-Fc were added at starting concentrations of 10 or 50 μg/mL, respectively, and incubated for 1 h at RT. Then, a HRP-conjugated, goat anti-human IgG antibody (AP113P, Merckmillipore) was added at a concentration of 1:10,000 and incubated for 1 h at RT. Finally, signal was revealed using a 1-Step Ultra TMB-ELISA Substrate Solution (12617087, Thermo Fisher) with the incubation of cSLAM-Fc and cLRP6-Fc for 1 and 10 min, respectively. The reactions were blocked via the addition of sulfuric acid (1 M). The optical density (OD) at 450 nm was measured with a microplate reader (Cytation 5; BioTek).

## References

[1] Lemos de Matos A, Franco LS, McFadden G. 2020. Oncolytic viruses and the immune system: the dynamic duo. Mol Ther Methods Clin Dev 17:349–358. doi:10.1016/j.omtm.2020.01.001. 32071927 PMC7015832

[2] Rahman MM, McFadden G. 2021. Oncolytic viruses: newest frontier for cancer immunotherapy. Cancers 13:5452. doi:10.3390/cancers13215452. 34771615 PMC8582515

[3] Johnson DB, Puzanov I, Kelley MC. 2015. Talimogene laherparepvec (T-VEC) for the treatment of advanced melanoma. Immunotherapy 7:611–619. doi:10.2217/imt.15.35. 26098919 PMC4519012

[4] Liang M. 2018. Oncorine, the world first oncolytic virus medicine and its update in China. Curr Cancer Drug Targets 18:171–176. doi:10.2174/1568009618666171129221503. 29189159

[5] Todo T, Ito H, Ino Y, Ohtsu H, Ota Y, Shibahara J, Tanaka M. 2022. Intratumoral oncolytic herpes virus G47Δ for residual or recurrent glioblastoma: a phase 2 trial. Nat Med 28:1630–1639. doi:10.1038/s41591-022-01897-x. 35864254 PMC9388376

[6] Engeland CE, Ungerechts G. 2021. Measles virus as an oncolytic immunotherapy. Cancers (Basel) 13:544. doi:10.3390/cancers13030544. 33535479 PMC7867054

[7] Dispenzieri A, Tong C, LaPlant B, Lacy MQ, Laumann K, Dingli D, Zhou Y, Federspiel MJ, Gertz MA, Hayman S, Buadi F, O'Connor M, Lowe VJ, Peng K-W, Russell SJ. 2017. Phase I trial of systemic administration of Edmonston strain of measles virus genetically engineered to express the sodium iodide symporter in patients with recurrent or refractory multiple myeloma. Leukemia 31:2791–2798. doi:10.1038/leu.2017.120. 28439108 PMC5656536

[8] Msaouel P, Opyrchal M, Dispenzieri A, Peng KW, Federspiel MJ, Russell SJ, Galanis E. 2018. Clinical trials with oncolytic measles virus: current status and future prospects. Curr Cancer Drug Targets 18:177–187. doi:10.2174/1568009617666170222125035. 28228086 PMC5630504

[9] Packiriswamy N, Upreti D, Zhou Y, Khan R, Miller A, Diaz RM, Rooney CM, Dispenzieri A, Peng K-W, Russell SJ. 2020. Oncolytic measles virus therapy enhances tumor antigen-specific T-cell responses in patients with multiple myeloma. Leukemia 34:3310–3322. doi:10.1038/s41375-020-0828-7. 32327728 PMC7581629

[10] von Messling V, Oezguen N, Zheng Q, Vongpunsawad S, Braun W, Cattaneo R. 2005. Nearby clusters of hemagglutinin residues sustain SLAM-dependent canine distemper virus entry in peripheral blood mononuclear cells. J Virol 79:5857–5862. doi:10.1128/JVI.79.9.5857-5862.2005. 15827201 PMC1082760

[11] Noyce RS, Richardson CD. 2012. Nectin 4 is the epithelial cell receptor for measles virus. Trends Microbiol 20:429–439. doi:10.1016/j.tim.2012.05.006. 22721863

[12] Noyce RS, Delpeut S, Richardson CD. 2013. Dog nectin-4 is an epithelial cell receptor for canine distemper virus that facilitates virus entry and syncytia formation. Virology 436:210–220. doi:10.1016/j.virol.2012.11.011. 23260107

[13] Dörig RE, Marcil A, Chopra A, Richardson CD. 1993. The human CD46 molecule is a receptor for measles virus (Edmonston strain). Cell 75:295–305. doi:10.1016/0092-8674(93)80071-l. 8402913

[14] Muñoz-Alía MÁ, Nace RA, Tischer A, Zhang L, Bah ES, Auton M, Russell SJ. 2021. MeV-Stealth: a CD46-specific oncolytic measles virus resistant to neutralization by measles-immune human serum. PLoS Pathog 17:e1009283. doi:10.1371/journal.ppat.1009283. 33534834 PMC7886131

[15] Rouxel RN, Svitek N, von Messling V. 2009. A chimeric measles virus with canine distemper envelope protects ferrets from lethal distemper challenge. Vaccine 27:4961–4966. doi:10.1016/j.vaccine.2009.05.096. 19540272

[16] Miest TS, Yaiw K-C, Frenzke M, Lampe J, Hudacek AW, Springfeld C, von Messling V, Ungerechts G, Cattaneo R. 2011. Envelope-chimeric entry-targeted measles virus escapes neutralization and achieves oncolysis. Mol Ther 19:1813–1820. doi:10.1038/mt.2011.92. 21610701 PMC3188758

[17] Muñoz-Alía MA, Russell SJ. 2019. Probing morbillivirus antisera neutralization using functional chimerism between measles virus and canine distemper virus envelope glycoproteins. Viruses 11:688. doi:10.3390/v11080688. 31357579 PMC6722617

[18] Green RG, Carlson WE. 1945. The immunization of foxes and dogs to distemper with ferret-passage virus. JAm Vet Med Ass: 131–142.

[19] Haig DA. 1956. Canine distemperimmunisation with avianised virus. Onderst J Vet Res: 19–53.

[20] Woma TY, van Vuuren M, Bosman A-M, Quan M, Oosthuizen M, Bwala DG, Ibu JO, Ularamu HG, Shamaki D. 2011. Genetic variant of canine distemper virus from clinical cases in vaccinated dogs in South Africa. Nig Vet J 31. doi:10.4314/nvj.v31i1.68938.

[21] Gröne A, Fonfara S, Baumgärtner W. 2002. Cell type-dependent cytokine expression after canine distemper virus infection. Viral Immunol 15:493–505. doi:10.1089/088282402760312368. 12479398

[22] Suter SE, Chein MB, von Messling V, Yip B, Cattaneo R, Vernau W, Madewell BR, London CA. 2005. In vitro canine distemper virus infection of canine lymphoid cells: a prelude to oncolytic therapy for lymphoma. Clin Cancer Res 11:1579–1587. doi:10.1158/1078-0432.CCR-04-1944. 15746063

[23] Shrestha N, Gall FM, Mathieu C, Hierweger MM, Brügger M, Alves MP, Vesin J, Banfi D, Kalbermatter D, Horvat B, Chambon M, Turcatti G, Fotiadis D, Riedl R, Plattet P. 2021. Highly potent host-specific small-molecule inhibitor of paramyxovirus and pneumovirus replication with high resistance barrier. mBio 12:e0262121. doi:10.1128/mBio.02621-21. 34724816 PMC8561388

[24] Han J, Perez JT, Chen C, Li Y, Benitez A, Kandasamy M, Lee Y, Andrade J, tenOever B, Manicassamy B. 2018. Genome-wide CRISPR/Cas9 screen identifies host factors essential for influenza virus replication. Cell Rep 23:596–607. doi:10.1016/j.celrep.2018.03.045. 29642015 PMC5939577

[25] Orchard RC, Wilen CB, Doench JG, Baldridge MT, McCune BT, Lee Y-CJ, Lee S, Pruett-Miller SM, Nelson CA, Fremont DH, Virgin HW. 2016. Discovery of a proteinaceous cellular receptor for a norovirus. Science 353:933–936. doi:10.1126/science.aaf1220. 27540007 PMC5484048

[26] Park RJ, Wang T, Koundakjian D, Hultquist JF, Lamothe-Molina P, Monel B, Schumann K, Yu H, Krupzcak KM, Garcia-Beltran W, Piechocka-Trocha A, Krogan NJ, Marson A, Sabatini DM, Lander ES, Hacohen N, Walker BD. 2017. A genome-wide CRISPR screen identifies a restricted set of HIV host dependency factors. Nat Genet 49:193–203. doi:10.1038/ng.3741. 27992415 PMC5511375

[27] Zhang R, Miner JJ, Gorman MJ, Rausch K, Ramage H, White JP, Zuiani A, Zhang P, Fernandez E, Zhang Q, Dowd KA, Pierson TC, Cherry S, Diamond MS. 2016. A CRISPR screen defines a signal peptide processing pathway required by flaviviruses. Nature 535:164–168. doi:10.1038/nature18625. 27383988 PMC4945490

[28] Wyss M, Gradauskaite V, Ebert N, Thiel V, Zurbriggen A, Plattet P. 2022. Efficient recovery of attenuated canine distemper virus from cDNA. Virus Res 316:198796. doi:10.1016/j.virusres.2022.198796. 35568090

[29] Van Leeuwen IS, Hellmèn E, Cornelisse CJ, Van den Burgh B, Rutteman GR. 1996. P53 mutations in mammary tumor cell lines and corresponding tumor tissues in the dog. Anticancer Res 16:3737–3744. 9042250

[30] Veillette A. 2010. SLAM-family receptors: immune regulators with or without SAP-family adaptors. Cold Spring Harb Perspect Biol 2:a002469. doi:10.1101/cshperspect.a002469. 20300214 PMC2829957

[31] Li W, Xu H, Xiao T, Cong L, Love MI, Zhang F, Irizarry RA, Liu JS, Brown M, Liu XS. 2014. MAGeCK enables robust identification of essential genes from genome-scale CRISPR/Cas9 knockout screens. Genome Biol 15:554. doi:10.1186/s13059-014-0554-4. 25476604 PMC4290824

[32] Hart T, Chandrashekhar M, Aregger M, Steinhart Z, Brown KR, MacLeod G, Mis M, Zimmermann M, Fradet-Turcotte A, Sun S, Mero P, Dirks P, Sidhu S, Roth FP, Rissland OS, Durocher D, Angers S, Moffat J. 2015. High-resolution CRISPR screens reveal fitness genes and genotype-specific cancer liabilities. Cell 163:1515–1526. doi:10.1016/j.cell.2015.11.015. 26627737

[33] Imkeller K, Ambrosi G, Boutros M, Huber W. 2020. gscreend: modelling asymmetric count ratios in CRISPR screens to decrease experiment size and improve phenotype detection. Genome Biol 21:53. doi:10.1186/s13059-020-1939-1. 32122365 PMC7052974

[34] Brinkman EK, Chen T, Amendola M, van Steensel B. 2014. Easy quantitative assessment of genome editing by sequence trace decomposition. Nucleic Acids Res 42:e168. doi:10.1093/nar/gku936. 25300484 PMC4267669

[35] Boudreau CE, York D, Higgins RJ, LeCouteur RA, Dickinson PJ. 2017. Molecular signalling pathways in canine gliomas. Vet Comp Oncol 15:133–150. doi:10.1111/vco.12147. 25808605

[36] York D, Higgins RJ, LeCouteur RA, Wolfe AN, Grahn R, Olby N, Campbell M, Dickinson PJ. 2012. TP53 mutations in canine brain tumors. Vet Pathol 49:796–801. doi:10.1177/0300985811424734. 22002975

[37] Moeschler S, Locher S, Conzelmann K-K, Krämer B, Zimmer G. 2016. Quantification of Lyssavirus-neutralizing antibodies using vesicular stomatitis virus pseudotype particles. Viruses 8:254. doi:10.3390/v8090254. 27649230 PMC5035968

[38] Rentsch MB, Zimmer G. 2011. A vesicular stomatitis virus replicon-based bioassay for the rapid and sensitive determination of multi-species type I interferon. PLoS One 6:e25858. doi:10.1371/journal.pone.0025858. 21998709 PMC3187809

[39] Yasui N, Mihara E, Nampo M, Tamura-Kawakami K, Unno H, Matsumoto K, Takagi J. 2010. Detection of endogenous LRP6 expressed on human cells by monoclonal antibodies specific for the native conformation. J Immunol Methods 352:153–160. doi:10.1016/j.jim.2009.11.016. 19945460

[40] Clevers H. 2006. Wnt/β-catenin signaling in development and disease. Cell 127:469–480. doi:10.1016/j.cell.2006.10.018. 17081971

[41] Li Y, Lu W, He X, Schwartz AL, Bu G. 2004. LRP6 expression promotes cancer cell proliferation and tumorigenesis by altering β-catenin subcellular distribution. Oncogene 23:9129–9135. doi:10.1038/sj.onc.1208123. 15516984

[42] Ruiz-Canada C, Kelleher DJ, Gilmore R. 2009. Cotranslational and posttranslational N-glycosylation of polypeptides by distinct mammalian OST isoforms. Cell 136:272–283. doi:10.1016/j.cell.2008.11.047. 19167329 PMC2859625

[43] Gradauskaite V, Khosravi M, Plattet P. 2022. Selective SLAM/CD150 receptor-detargeting of canine distemper virus. Virus Res 318:198841. doi:10.1016/j.virusres.2022.198841. 35649483

[44] Zipperle L, Langedijk JPM, Orvell C, Vandevelde M, Zurbriggen A, Plattet P. 2010. Identification of key residues in virulent canine distemper virus hemagglutinin that control CD150/SLAM-binding activity. J Virol 84:9618–9624. doi:10.1128/JVI.01077-10. 20631152 PMC2937642

[45] Avila M, Alves L, Khosravi M, Ader-Ebert N, Origgi F, Schneider-Schaulies J, Zurbriggen A, Plemper RK, Plattet P. 2014. Molecular determinants defining the triggering range of prefusion F complexes of canine distemper virus. J Virol 88:2951–2966. doi:10.1128/JVI.03123-13. 24371057 PMC3958098

[46] Sun A, Prussia A, Zhan W, Murray EE, Doyle J, Cheng L-T, Yoon J-J, Radchenko EV, Palyulin VA, Compans RW, Liotta DC, Plemper RK, Snyder JP. 2006. Nonpeptide inhibitors of measles virus entry. J Med Chem 49:5080–5092. doi:10.1021/jm0602559. 16913698

[47] Puff C, Krudewig C, Imbschweiler I, Baumgärtner W, Alldinger S. 2009. Influence of persistent canine distemper virus infection on expression of RECK, matrix-metalloproteinases and their inhibitors in a canine macrophage/monocytic tumour cell line (DH82). Vet J 182:100–107. doi:10.1016/j.tvjl.2008.03.026. 18684651

[48] Pfankuche VM, Spitzbarth I, Lapp S, Ulrich R, Deschl U, Kalkuhl A, Baumgärtner W, Puff C. 2017. Reduced angiogenic gene expression in morbillivirus-triggered oncolysis in a translational model for histiocytic sarcoma. J Cell Mol Med 21:816–830. doi:10.1111/jcmm.13023. 27860224 PMC5345635

[49] Zhao J, Ren Y, Chen J, Zheng J, Sun D. 2020. Viral pathogenesis, recombinant vaccines, and oncolytic virotherapy: applications of the canine distemper virus reverse genetics system. Viruses 12:339. doi:10.3390/v12030339. 32244946 PMC7150803

[50] von Messling V, Springfeld C, Devaux P, Cattaneo R. 2003. A ferret model of canine distemper virus virulence and immunosuppression. J Virol 77:12579–12591. doi:10.1128/jvi.77.23.12579-12591.2003. 14610181 PMC262577

[51] Herz J, Strickland DK. 2001. LRP: a multifunctional scavenger and signaling receptor. J Clin Invest 108:779–784. doi:10.1172/JCI13992. 11560943 PMC200939

[52] Finkelshtein D, Werman A, Novick D, Barak S, Rubinstein M. 2013. LDL receptor and its family members serve as the cellular receptors for vesicular stomatitis virus. Proc Natl Acad Sci USA 110:7306–7311. doi:10.1073/pnas.1214441110. 23589850 PMC3645523

[53] Kounnas MZ, Morris RE, Thompson MR, FitzGerald DJ, Strickland DK, Saelinger CB. 1992. The alpha 2-macroglobulin receptor/low density lipoprotein receptor-related protein binds and internalizes Pseudomonas exotoxin A. J Biol Chem 267:12420–12423. doi:10.1016/S0021-9258(18)42291-0. 1618748

[54] Wei W, Lu Q, Chaudry GJ, Leppla SH, Cohen SN. 2006. The LDL receptor-related protein LRP6 mediates internalization and lethality of anthrax toxin. Cell 124:1141–1154. doi:10.1016/j.cell.2005.12.045. 16564009

[55] Tachibana M, Holm M-L, Liu C-C, Shinohara M, Aikawa T, Oue H, Yamazaki Y, Martens YA, Murray ME, Sullivan PM, Weyer K, Glerup S, Dickson DW, Bu G, Kanekiyo T. 2019. APOE4-mediated amyloid-β pathology depends on its neuronal receptor LRP1. J Clin Invest 129:1272–1277. doi:10.1172/JCI124853. 30741718 PMC6391135

[56] Deane R, Wu Z, Sagare A, Davis J, Du Yan S, Hamm K, Xu F, Parisi M, LaRue B, Hu HW, Spijkers P, Guo H, Song X, Lenting PJ, Van Nostrand WE, Zlokovic BV. 2004. LRP/amyloid β-peptide interaction mediates differential brain efflux of Aβ isoforms. Neuron 43:333–344. doi:10.1016/j.neuron.2004.07.017. 15294142

[57] Ganaie SS, Schwarz MM, McMillen CM, Price DA, Feng AX, Albe JR, Wang W, Miersch S, Orvedahl A, Cole AR, Sentmanat MF, Mishra N, Boyles DA, Koenig ZT, Kujawa MR, Demers MA, Hoehl RM, Moyle AB, Wagner ND, Stubbs SH, Cardarelli L, Teyra J, McElroy A, Gross ML, Whelan SPJ, Doench J, Cui X, Brett TJ, Sidhu SS, Virgin HW, Egawa T, Leung DW, Amarasinghe GK, Hartman AL. 2021. Lrp1 is a host entry factor for Rift Valley fever virus. Cell 184:5163–5178.e24. doi:10.1016/j.cell.2021.09.001. 34559985 PMC8786218

[58] Schwarz MM, Price DA, Ganaie SS, Feng A, Mishra N, Hoehl RM, Fatma F, Stubbs SH, Whelan SPJ, Cui X, Egawa T, Leung DW, Amarasinghe GK, Hartman AL. 2022. Oropouche orthobunyavirus infection is mediated by the cellular host factor Lrp1. Proc Natl Acad Sci USA 119:e2204706119. doi:10.1073/pnas.2204706119. 35939689 PMC9388146

[59] Ma H, Kim AS, Kafai NM, Earnest JT, Shah A, Case JB, Basore K, Gilliland TC, Sun C, Nelson CA, Thackray LB, Klimstra WB, Fremont DH, Diamond MS. 2020. LDLRAD3 is a receptor for Venezuelan equine encephalitis virus. Nature 588:308–314. doi:10.1038/s41586-020-2915-3. 33208938 PMC7769003

[60] Devignot S, Sha TW, Burkard T, Schmerer P, Hagelkruys A, Mirazimi A, Elling U, Penninger JM, Weber F. 2022. Low density lipoprotein receptor-related protein 1 (LRP1) is a host factor for RNA viruses including SARS-CoV-2. 10.26508/lsa.202302005 PMC10114362 37072184

[61] Ren Q, Chen J, Liu Y. 2021. LRP5 and LRP6 in Wnt signaling: similarity and divergence. Front Cell Dev Biol 9:670960. doi:10.3389/fcell.2021.670960. 34026761 PMC8134664

[62] Wang Z-M, Luo J-Q, Xu L-Y, Zhou H-H, Zhang W. 2018. Harnessing low-density lipoprotein receptor protein 6 (LRP6) genetic variation and Wnt signaling for innovative diagnostics in complex diseases. Pharmacogenomics J 18:351–358. doi:10.1038/tpj.2017.28. 28696417

[63] Tatsuo H, Okuma K, Tanaka K, Ono N, Minagawa H, Takade A, Matsuura Y, Yanagi Y. 2000. Virus entry is a major determinant of cell tropism of Edmonston and wild-type strains of measles virus as revealed by vesicular stomatitis virus pseudotypes bearing their envelope proteins. J Virol 74:4139–4145. doi:10.1128/jvi.74.9.4139-4145.2000. 10756026 PMC111928

[64] Bankamp B, Hodge G, McChesney MB, Bellini WJ, Rota PA. 2008. Genetic changes that affect the virulence of measles virus in a rhesus macaque model. Virology 373:39–50. doi:10.1016/j.virol.2007.11.025. 18155263

[65] Cheng Z, Biechele T, Wei Z, Morrone S, Moon RT, Wang L, Xu W. 2011. Crystal structures of the extracellular domain of LRP6 and its complex with DKK1. Nat Struct Mol Biol 18:1204–1210. doi:10.1038/nsmb.2139. 21984209 PMC3249237

[66] Matoba K, Mihara E, Tamura-Kawakami K, Miyazaki N, Maeda S, Hirai H, Thompson S, Iwasaki K, Takagi J. 2017. Conformational freedom of the LRP6 ectodomain is regulated by N-glycosylation and the binding of the Wnt antagonist Dkk1. Cell Rep 18:32–40. doi:10.1016/j.celrep.2016.12.017. 28052259

[67] Tung EK-K, Wong BY-C, Yau T-O, Ng IO-L. 2012. Upregulation of the Wnt co-receptor LRP6 promotes hepatocarcinogenesis and enhances cell invasion. PLoS One 7:e36565. doi:10.1371/journal.pone.0036565. 22570728 PMC3343020

[68] Ma J, Lu W, Chen D, Xu B, Li Y. 2017. Role of Wnt co-receptor LRP6 in triple negative breast cancer cell migration and invasion. J Cell Biochem 118:2968–2976. doi:10.1002/jcb.25956. 28247948 PMC10928515

[69] Yao Q, An Y, Hou W, Cao Y-N, Yao M-F, Ma N-N, Hou L, Zhang H, Liu H-J, Zhang B. 2017. LRP6 promotes invasion and metastasis of colorectal cancer through cytoskeleton dynamics. Oncotarget 8:109632–109645. doi:10.18632/oncotarget.22759. 29312635 PMC5752548

[70] Rismani E, Fazeli MS, Mahmoodzadeh H, Movassagh A, Azami S, Karimipoor M, Teimoori-Toolabi L. 2017. Pattern of LRP6 gene expression in tumoral tissues of colorectal cancer. Cancer Biomark 19:151–159. doi:10.3233/CBM-160175. 28387660 PMC13020720

[71] Liu C-C, Prior J, Piwnica-Worms D, Bu G. 2010. LRP6 overexpression defines a class of breast cancer subtype and is a target for therapy. Proc Natl Acad Sci USA 107:5136–5141. doi:10.1073/pnas.0911220107. 20194742 PMC2841938

[72] Matveeva OV, Chumakov PM. 2018. Defects in interferon pathways as potential biomarkers of sensitivity to oncolytic viruses. Rev Med Virol 28:e2008. doi:10.1002/rmv.2008. 30209859 PMC6906582

[73] Buchholz UJ, Finke S, Conzelmann K-K. 1999. Generation of bovine respiratory syncytial virus (BRSV) from cDNA: BRSV NS2 is not essential for virus replication in tissue culture, and the human RSV leader region acts as a functional BRSV genome promoter. J Virol 73:251–259. doi:10.1128/JVI.73.1.251-259.1999. 9847328 PMC103829

[74] Cherpillod P, Beck K, Zurbriggen A, Wittek R. 1999. Sequence analysis and expression of the attachment and fusion proteins of canine distemper virus wild-type strain A75/17. J Virol 73:2263–2269. doi:10.1128/JVI.73.3.2263-2269.1999. 9971809 PMC104471

[75] Khosravi M, Bringolf F, Röthlisberger S, Bieringer M, Schneider-Schaulies J, Zurbriggen A, Origgi F, Plattet P. 2016. Canine distemper virus fusion activation: critical role of residue E123 of CD150/SLAM. J Virol 90:1622–1637. doi:10.1128/JVI.02405-15. 26608324 PMC4719633

[76] Torriani G, Trofimenko E, Mayor J, Fedeli C, Moreno H, Michel S, Heulot M, Chevalier N, Zimmer G, Shrestha N, Plattet P, Engler O, Rothenberger S, Widmann C, Kunz S. 2019. Identification of clotrimazole derivatives as specific inhibitors of arenavirus fusion. J Virol 93. doi:10.1128/JVI.01744-18. PMC6401469 30626681

[77] 2022. Biparatopic sybodies neutralize SARS-CoV-2 variants of concern and mitigate drug resistance. EMBO Rep 23:e54199. 35253970 10.15252/embr.202154199 PMC8982573

[78] Shrestha N, Gall FM, Vesin J, Chambon M, Turcatti G, Fotiadis D, Riedl R, Plattet P. 2021. Antiviral screen against canine distemper virus-induced membrane fusion activity. Viruses 13:128. doi:10.3390/v13010128. 33477492 PMC7831055

[79] Logan N, McMonagle E, Drew AA, Takahashi E, McDonald M, Baron MD, Gilbert M, Cleaveland S, Haydon DT, Hosie MJ, Willett BJ. 2016. Efficient generation of vesicular stomatitis virus (VSV)-pseudotypes bearing morbilliviral glycoproteins and their use in quantifying virus neutralising antibodies. Vaccine 34:814–822. doi:10.1016/j.vaccine.2015.12.006. 26706278 PMC4742518

[80] Zettl F, Meister TL, Vollmer T, Fischer B, Steinmann J, Krawczyk A, V’kovski P, Todt D, Steinmann E, Pfaender S, Zimmer G. 2020. Rapid quantification of SARS-CoV-2-neutralizing antibodies using propagation-defective vesicular stomatitis virus pseudotypes. Vaccines 8:386. doi:10.3390/vaccines8030386. 32679691 PMC7563800

