# Supplementary material for: LRP6 Is a Functional Receptor for Attenuated Canine Distemper Virus
Source: mBio. 2023 Jan 16;14(1):e03114-22. doi: 10.1128/mbio.03114-22 (PMC9973313; doi:10.1128/mbio.03114-22)
Supplement: TABLE S2 [file mbio.03114-22-s0005.docx]

**Table S2: List of the first hundred ranked gene candidates identified in 2 experiments of the CRISPR/Cas9 screen. Analysis run with Mageck software, CDV treated group versus control at D15.**

| **ID** | **p-value** | **FDR** | **good_sgrna** | **LFC** |
| --- | --- | --- | --- | --- |
| LRP6 | 2.61E-06 | 0.037129 | 2 | 0.70676 |
| STT3A | 3.56E-06 | 0.037129 | 3 | 0.95896 |
| TMTC4 | 0.000101 | 0.450495 | 3 | 0.5839 |
| FAM129B | 0.000142 | 0.450495 | 3 | 0.62857 |
| RAN | 0.000143 | 0.450495 | 2 | 0.056397 |
| TIGIT | 0.000151 | 0.450495 | 2 | 0.46252 |
| UTRN | 0.000218 | 0.509901 | 3 | 0.63495 |
| EFCAB7 | 0.000269 | 0.561881 | 2 | 0.71568 |
| ZNF281 | 0.000424 | 0.710967 | 3 | 0.55721 |
| C1QBP | 0.000443 | 0.710967 | 2 | 0.41863 |
| OSTC | 0.000501 | 0.746464 | 3 | 0.64405 |
| WDR78 | 0.000584 | 0.775155 | 3 | 0.54524 |
| ZBTB7A | 0.000674 | 0.775155 | 2 | 0.634 |
| ADAM21 | 0.000695 | 0.775155 | 2 | 0.44141 |
| LYPD4 | 0.00078 | 0.775155 | 2 | 0.53507 |
| GNA13 | 0.000825 | 0.775155 | 2 | 0.073496 |
| NANP | 0.001042 | 0.775155 | 2 | 0.67968 |
| ACTR6 | 0.001109 | 0.775155 | 2 | 0.34796 |
| MLLT10 | 0.001145 | 0.775155 | 2 | 0.39584 |
| MET | 0.001158 | 0.775155 | 2 | 0.4009 |
| RPL17 | 0.001258 | 0.775155 | 2 | 0.50102 |
| MAP4K4 | 0.001294 | 0.775155 | 2 | 0.54639 |
| ST8SIA1 | 0.001365 | 0.775155 | 2 | 0.54161 |
| C5H11orf52 | 0.001417 | 0.775155 | 2 | 0.39582 |
| LOC610887 | 0.001426 | 0.775155 | 2 | 0.50069 |
| LOC100684561 | 0.001435 | 0.775155 | 2 | 0.32679 |
| ARID1B | 0.001465 | 0.775155 | 2 | 0.47348 |
| LOC111090006 | 0.001496 | 0.775155 | 2 | 0.49258 |
| FASN | 0.001542 | 0.775155 | 2 | 0.4289 |
| COG4 | 0.001597 | 0.775155 | 2 | 0.33347 |
| LOC475706 | 0.001749 | 0.811111 | 2 | 0.21674 |
| LOC100682600 | 0.001939 | 0.86107 | 2 | 0.57624 |
| ACVR1 | 0.002096 | 0.868278 | 2 | 0.61089 |
| UBR7 | 0.002103 | 0.868278 | 2 | 0.58368 |
| DIS3 | 0.002328 | 0.873191 | 2 | 0.22911 |
| TMEM242 | 0.002338 | 0.873191 | 2 | 0.4327 |
| RNASE4 | 0.002452 | 0.873191 | 2 | 0.22185 |
| ZFP1 | 0.002459 | 0.873191 | 2 | 0.5109 |
| CCDC30 | 0.002643 | 0.873191 | 2 | 0.36091 |
| KANSL2 | 0.002654 | 0.873191 | 2 | 0.36226 |
| BLOC1S3 | 0.002693 | 0.873191 | 2 | 0.4209 |
| LOC607692 | 0.00272 | 0.873191 | 2 | 0.36539 |
| RTKN | 0.002913 | 0.873521 | 2 | 0.34629 |
| GAB1 | 0.003107 | 0.873521 | 2 | 0.45587 |
| CCNT1 | 0.003123 | 0.873521 | 2 | 0.43008 |
| PPP4R3B | 0.003237 | 0.873521 | 2 | 0.58194 |
| STT3B | 0.003359 | 0.873521 | 2 | 0.53457 |
| GRIN2D | 0.003367 | 0.873521 | 2 | 0.55306 |
| GINM1 | 0.003433 | 0.873521 | 2 | 0.33904 |
| NOL4 | 0.003619 | 0.888585 | 2 | 0.24066 |
| PRSS55 | 0.003841 | 0.894479 | 2 | 0.47767 |
| TMEM165 | 0.003882 | 0.894479 | 2 | 0.30423 |
| BTD | 0.003935 | 0.894479 | 2 | 0.46146 |
| ATP8B3 | 0.003944 | 0.894479 | 2 | 0.37812 |
| LOC100688648 | 0.004125 | 0.903205 | 2 | 0.33923 |
| MINK1 | 0.004351 | 0.903205 | 2 | 0.48699 |
| BRSK1 | 0.004446 | 0.903205 | 2 | 0.33941 |
| TMED2 | 0.004481 | 0.903205 | 2 | 0.36737 |
| PSMG4 | 0.004521 | 0.903205 | 2 | 0.51751 |
| RHO | 0.00455 | 0.903205 | 2 | 0.45768 |
| NIFK | 0.004669 | 0.903205 | 2 | 0.26408 |
| CRYBG3 | 0.004731 | 0.903205 | 2 | 0.38303 |
| RWDD2A | 0.004856 | 0.903205 | 2 | 0.36492 |
| SOCS3 | 0.004875 | 0.903205 | 2 | 0.33005 |
| DDX4 | 0.004907 | 0.903205 | 2 | 0.2393 |
| DVL3 | 0.004915 | 0.903205 | 2 | 0.34817 |
| AMH | 0.005335 | 0.922737 | 2 | 0.43627 |
| INTS12 | 0.005353 | 0.922737 | 2 | 0.38316 |
| UFC1 | 0.005708 | 0.922737 | 2 | 0.51786 |
| LTC4S | 0.005714 | 0.922737 | 2 | 0.005006 |
| LRRC3B | 0.005782 | 0.922737 | 2 | 0.38402 |
| SDHAF3 | 0.005815 | 0.922737 | 2 | 0.55177 |
| RINT1 | 0.005826 | 0.922737 | 2 | 0.46007 |
| EMSY | 0.005869 | 0.922737 | 2 | 0.28405 |
| CCT3 | 0.005892 | 0.922737 | 2 | 0.33113 |
| SPG21 | 0.006002 | 0.922737 | 2 | 0.47557 |
| BCR | 0.006088 | 0.922737 | 2 | 0.35356 |
| LOC100686929 | 0.0061 | 0.922737 | 2 | 0.40648 |
| ZEB1 | 0.006173 | 0.922737 | 2 | 0.30382 |
| SLC39A10 | 0.006322 | 0.930312 | 2 | 0.6034 |
| ST8SIA4 | 0.006375 | 0.930312 | 2 | 0.41933 |
| STRA6 | 0.006717 | 0.935161 | 2 | 0.26913 |
| AFF3 | 0.006724 | 0.935161 | 2 | 0.21847 |
| LOC102153893 | 0.006846 | 0.935161 | 2 | 0.30702 |
| LOC106559263 | 0.006852 | 0.935161 | 2 | 0.27808 |
| LOC491373 | 0.006887 | 0.935161 | 2 | 0.45058 |
| FAT4 | 0.0069 | 0.935161 | 2 | 0.099452 |
| FABP5 | 0.007015 | 0.936991 | 2 | 0.36274 |
| SKIDA1 | 0.007086 | 0.936991 | 2 | 0.3516 |
| FAM118B | 0.007185 | 0.937407 | 2 | 0.3954 |
| FAM120B | 0.00735 | 0.940576 | 2 | 0.42434 |
| SUFU | 0.00743 | 0.940576 | 2 | 0.44217 |
| HACD3 | 0.00784 | 0.940576 | 2 | 0.52745 |
| ALCAM | 0.007923 | 0.940576 | 2 | 0.51708 |
| LOC100687054 | 0.007963 | 0.940576 | 2 | 0.2711 |
| CELSR2 | 0.00807 | 0.940576 | 2 | 0.47786 |
| MBNL1 | 0.00813 | 0.940576 | 2 | 0.52862 |
| IGFBP6 | 0.008206 | 0.940576 | 2 | 0.40587 |
| LRP5 | 0.008222 | 0.940576 | 2 | 0.3967 |
| LOC100856444 | 0.008281 | 0.940576 | 2 | 0.51748 |
