# Supplementary figures and images for: LRP6 Is a Functional Receptor for Attenuated Canine Distemper Virus
Source: mBio. 2023 Jan 16;14(1):e03114-22. doi: 10.1128/mbio.03114-22 (PMC9973313; doi:10.1128/mbio.03114-22)

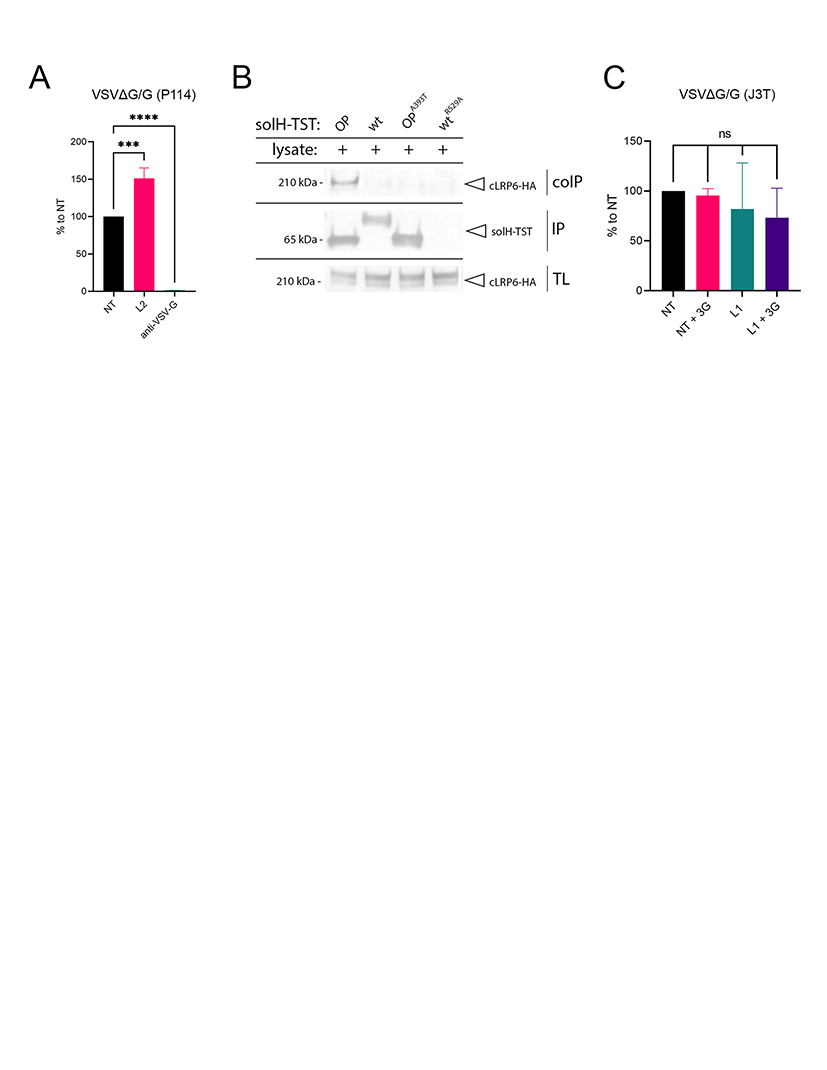

Supplement: FIG S3 [file mbio.03114-22-s0003.tif]

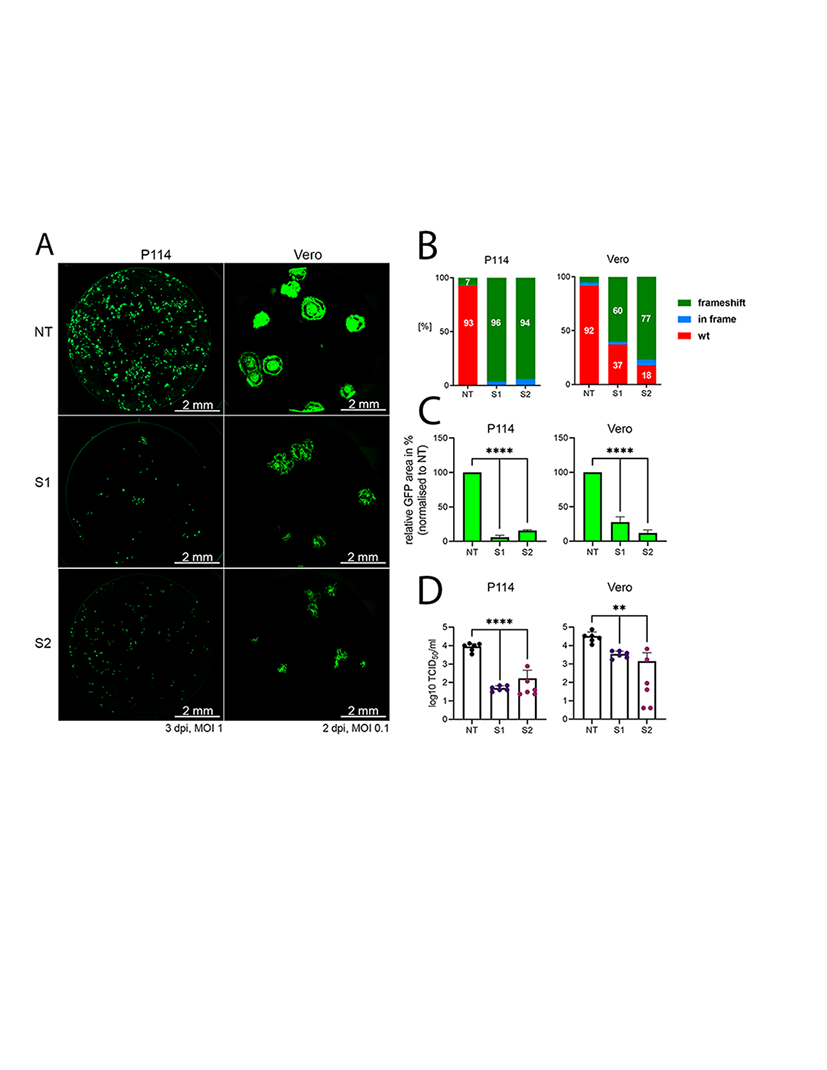

Supplement: FIG S1 [file mbio.03114-22-s0001.tif]

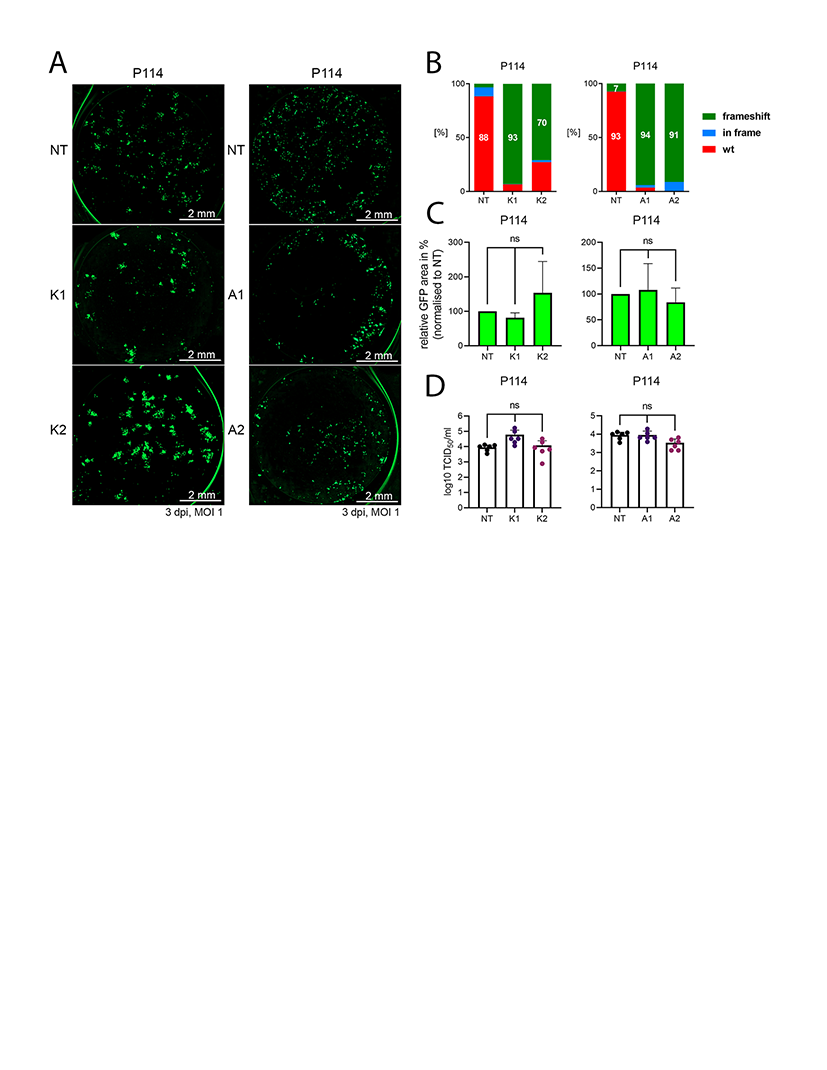

Supplement: FIG S2 [file mbio.03114-22-s0002.tif]
