# Supplementary material for: LRP6 Is a Functional Receptor for Attenuated Canine Distemper Virus
Source: mBio. 2023 Jan 16;14(1):e03114-22. doi: 10.1128/mbio.03114-22 (PMC9973313; doi:10.1128/mbio.03114-22)
Supplement: TABLE S3 [file mbio.03114-22-s0006.docx]

**Table S3: False discovery rate (FDR) and Log Fold Change (LFC) obtained after analysis (with MAGeCK or gscreend) of the CDV treated group (D15) compared to D0 with two experiments.**

|  | **LRP6** | | **STT3A** | | **ALCAM** | | **KCNE5** | |
| --- | --- | --- | --- | --- | --- | --- | --- | --- |
|  | **FDR** | **LFC** | **FDR** | **LFC** | **FDR** | **LFC** | **FDR** | **LFC** |
| **MAGeCK** | 0.002475 | 0.66982 | 0.540937 | 0.62645 | 0.607348 | 0.63824 | 0.540937 | 0.59581 |
| **gscreend** | 0 | 2.664975 | 0.075 | 1.953282 | 0.943139 | 1.466006 | 0.49 | 1.763745 |
